# Supplementary material for: Identification and establishment of type IV interferon and the characterization of interferon-υ including its class II cytokine receptors IFN-υR1 and IL-10R2
Source: Nat Commun. 2022 Feb 22;13:999. doi: 10.1038/s41467-022-28645-6 (PMC8863823; doi:10.1038/s41467-022-28645-6)
Supplement: Supplementary file 1 — Supplementary Information [file 41467_2022_28645_MOESM1_ESM.pdf]

Identification and establishment of type IV interferons and the characterization of interferon-υ including its class II cytokine receptors IFN-υR1 and IL-10R2

Supplementary Figures and Tables

Supplementary Figures 1-29
Supplementary Tables 1-6

Supplementary Figure 1

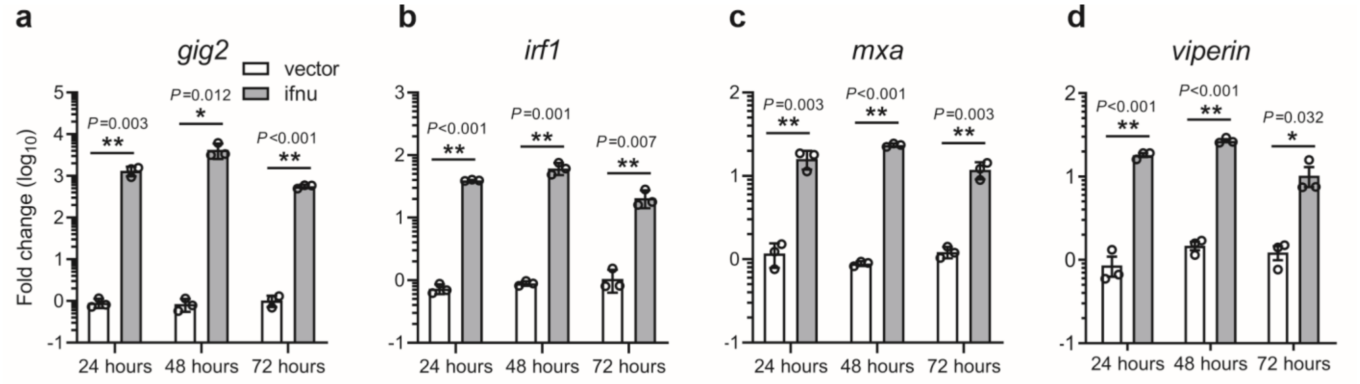

Supplementary Fig. 1. Induction of antiviral ISGs in IFN-υ-overexpressed zebrafish. Embryos (n = 150) were injected at one-cell stage with IFN-υ or empty vector plasmid, and after 24 hours, the mRNA level of ISGs, including *gig2* (a), *irf1* (b), *mxa* (c) and *viperin* (d), was detected by quantitative RT-PCR. The expression of the selected genes was normalized against *gapdh* and fold changes were calculated relative to control group (empty vector). Data represent mean ± SEM from three independent experiments. The two-tailed Student's *t*-test was used to determine the statistical significance, with * indicating $P < 0.05$, and ** $P < 0.01$.

Supplementary Figure 2

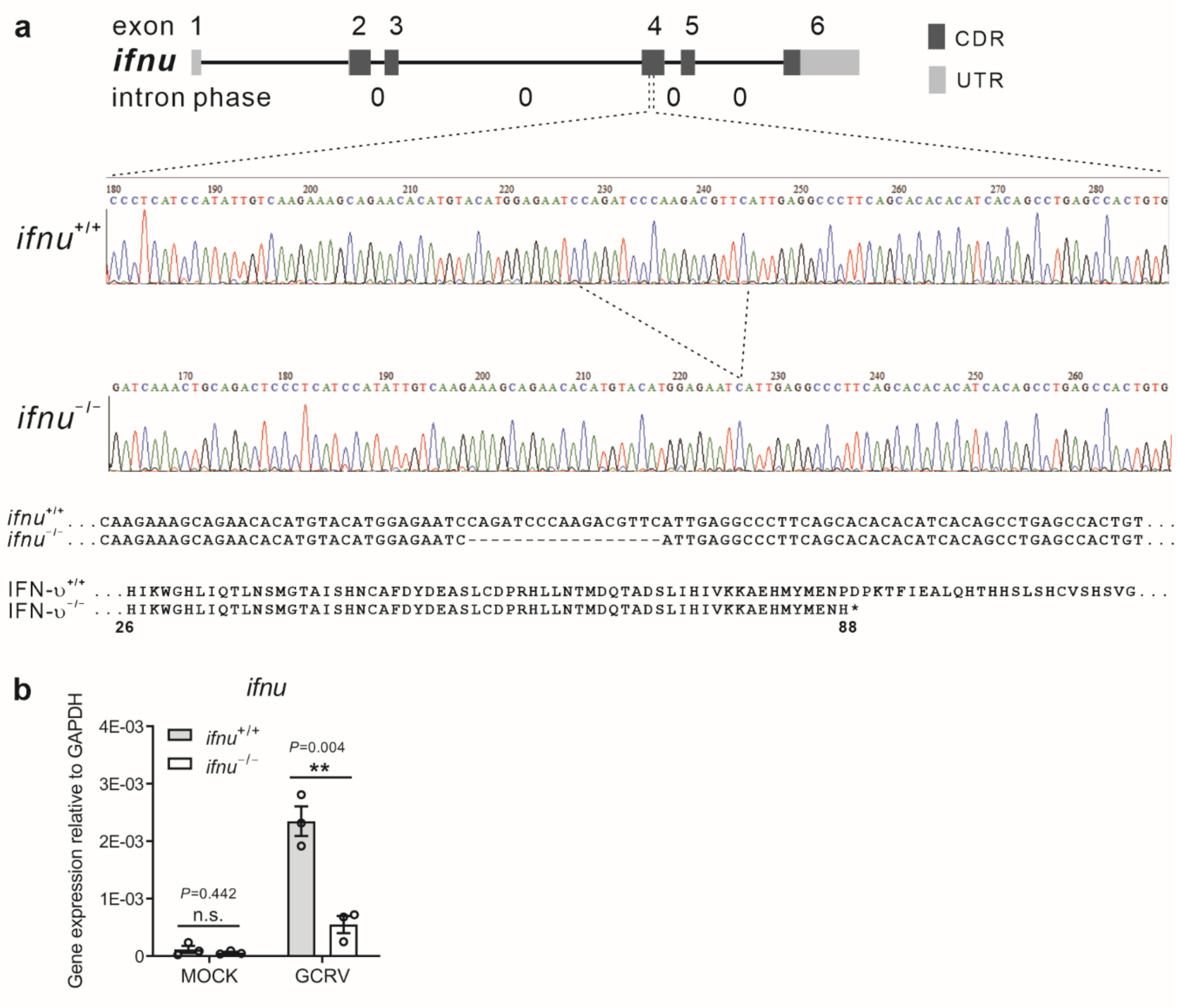

Supplementary Fig. 2. Generation of *ifnu* deficiency zebrafish. (a) The target site and sequence information in *ifnu* mutants. Cas9/gRNA system specific to *ifnu* targets on Exon 4 and leads to 17 bp deletion (5-CAGATCCCAAGACGTTC-3), which results in frameshift mutation and premature translation termination to generate a truncated protein with 88 aa. (b) *ifnu* expression in deficiency zebrafish. Zebrafish larvae (5 dpf, n = 33) were infected with GCRV for 24 hours and were collected to extract RNA to determine the expression of *ifnu*, which was normalized against *gapdh* by quantitative RT-PCR. The word, MOCK, represents the zebrafish larvae (6 dpf, n = 33) which were not infected with the virus. Data represent mean ± SEM from three independent experiments. The two-tailed Student's *t*-test was used to determine the statistical significance, * indicating $P < 0.05$, and ** $P < 0.01$.

Supplementary Figure 3

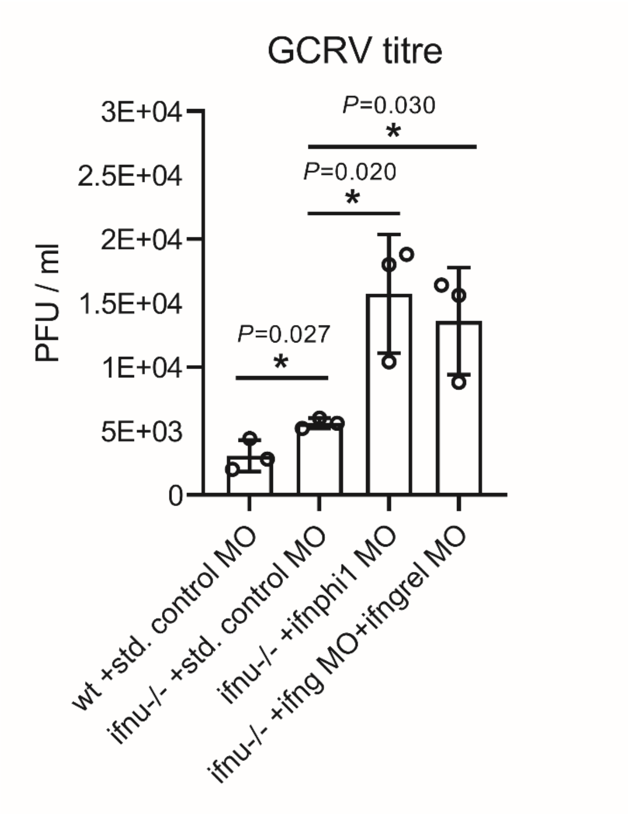

Supplementary Fig. 3. Effects on viral titre in response to GCRV infection by knockdown of IFN-φ1, IFN-γ and IFN-γrel in *ifnu* deficiency zebrafish. Embryos at one-cell stage (n = 150) were micro-injected with the IFN morpholinos or standard (std) control morpholino for 72 hours, and hatched zebrafish larvae (n = 33) were infected with GCRV for 24 hours and then collected to detect viral titers. Data represent mean ± SEM from three independent experiments. The two-tailed Student's *t*-test was used to determine the statistical significance, with * indicating $P < 0.05$, and ** $P < 0.01$.

Supplementary Figure 4

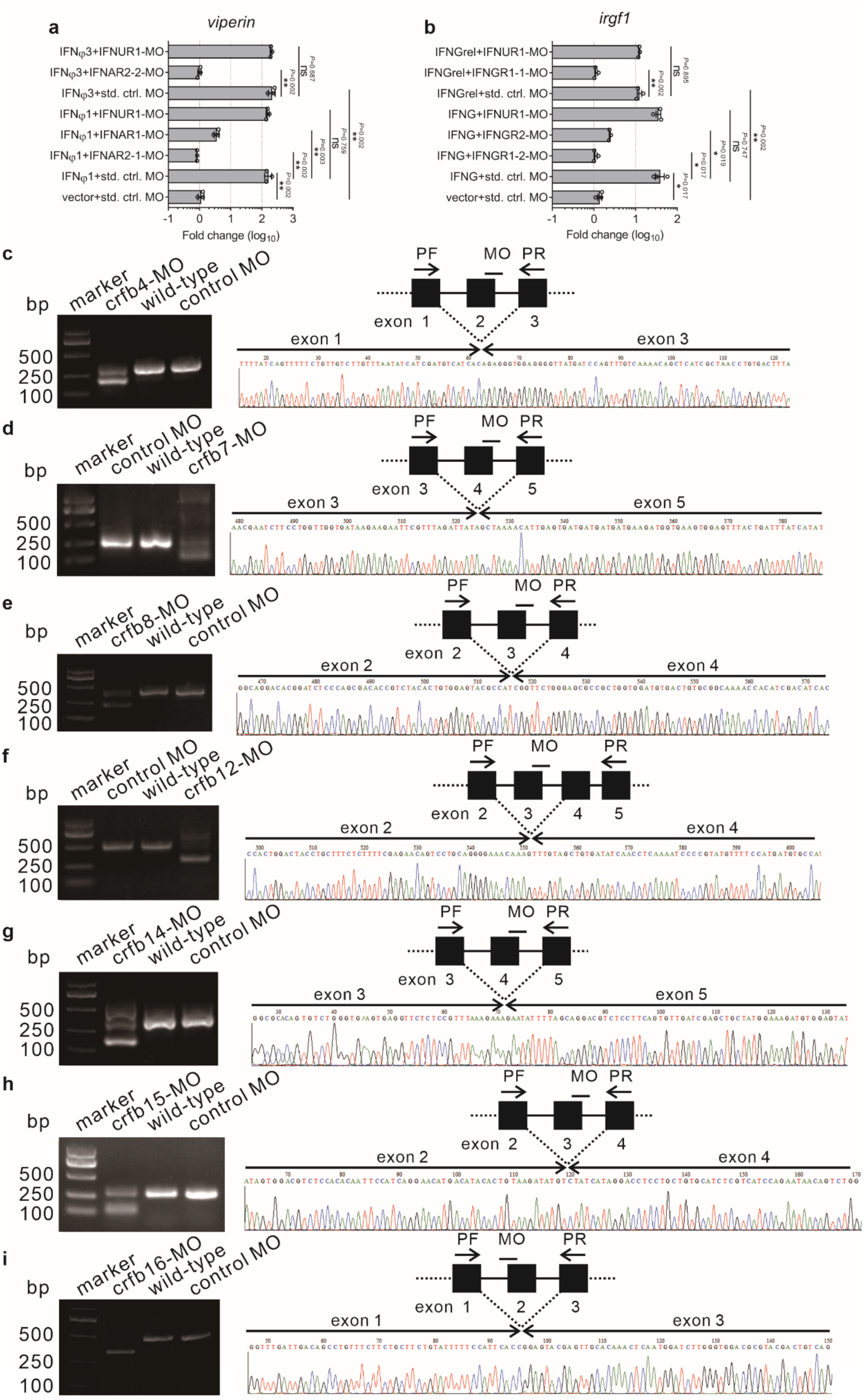

Supplementary Fig. 4. Knockdown test of CRFBs in zebrafish. Inhibitory effects of zebrafish IFN possible receptors' morpholinos (MOs) on *viperin* (a) or *irgf1* (b) expression induced by IFN1 (IFN-φ1) / IFN3 (IFN-φ3) or IFN-γ (IFN-γ2) / IFN-γrel (IFN-γ1), respectively. IFN plasmids and different MOs were co-injected into one-cell stage embryos (n = 150), and at 2 dpf the mRNA level of *viperin* or *irgf1* was detected by quantitative RT-PCR. The expression of selected genes was normalized against *gapdh*, and fold changes were calculated relative to control group which were co-injected into embryos with empty plasmid and standard control MOs. Data represent mean ± SEM from three independent experiments. The two-tailed Student's *t*-test was used to determine the statistical significance, with * indicating $P < 0.05$ and ** indicating $P < 0.01$. Knockdown effect and strategy for the *crfb4*-MO (c), *crfb7*-MO (d), *crfb8*-MO (e), *crfb12*-MO (f), *crfb14*-MO (g), *crfb15*-MO (h) and *crfb16*-MO (i). After the injection of *crfb*-MOs for 48 hours, RT-PCRs and sequencing of PCR production were performed to detect knockdown effect of different MOs. Arrows and solid lines represent PCR primers (Supplementary Table 3) and MOs, respectively. Data represent two independent experiments. All MOs described above have been proved previously to possess knockdown activity[1-3].

Supplementary Figure 5

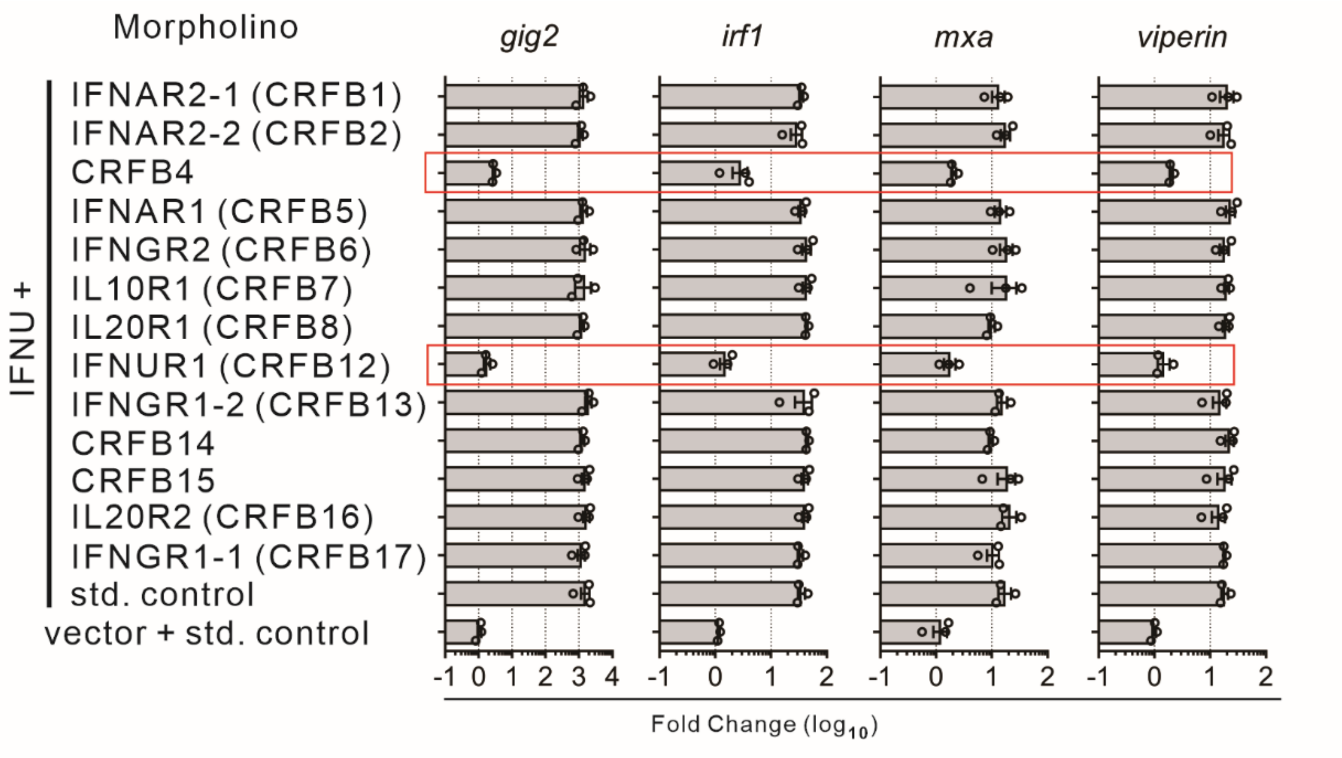

Supplementary Fig. 5. Effects on IFN-υ-induced ISG expression by knockdown of CRFB morpholinos in zebrafish at 24 hpf. IFN-υ expressing plasmid and different morpholinos were co-injected into zebrafish embryos (n = 500) at one-cell stage, and 24 hours later the expression of ISGs was detected by quantitative RT-PCR. The expression of the selected genes was normalized against *gapdh* and fold changes were calculated relative to control group which was co-injected in embryos with empty plasmid and standard (std.) control morpholinos. Data represent mean ± SEM from three independent experiments.

Supplementary Figure 6

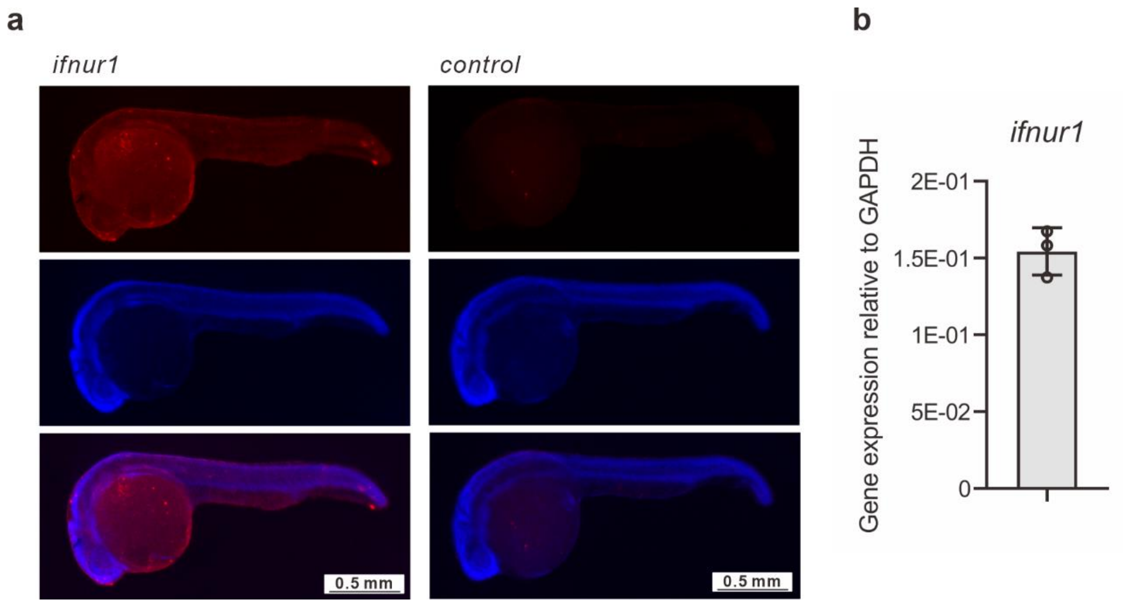

Supplementary Fig. 6. Expressional pattern of *ifnur1* in zebrafish embryos. (a) Detection of *ifnur1* expression by whole-mount *in situ* hybridization (WISH). Data represent two independent experiments. (b) Expression of *ifnur1* was detected by quantitative RT-PCR. The expression level of *ifnur1* was calculated relative to *gapdh*. Data represent mean ± SEM from three independent experiments.

Supplementary Figure 7

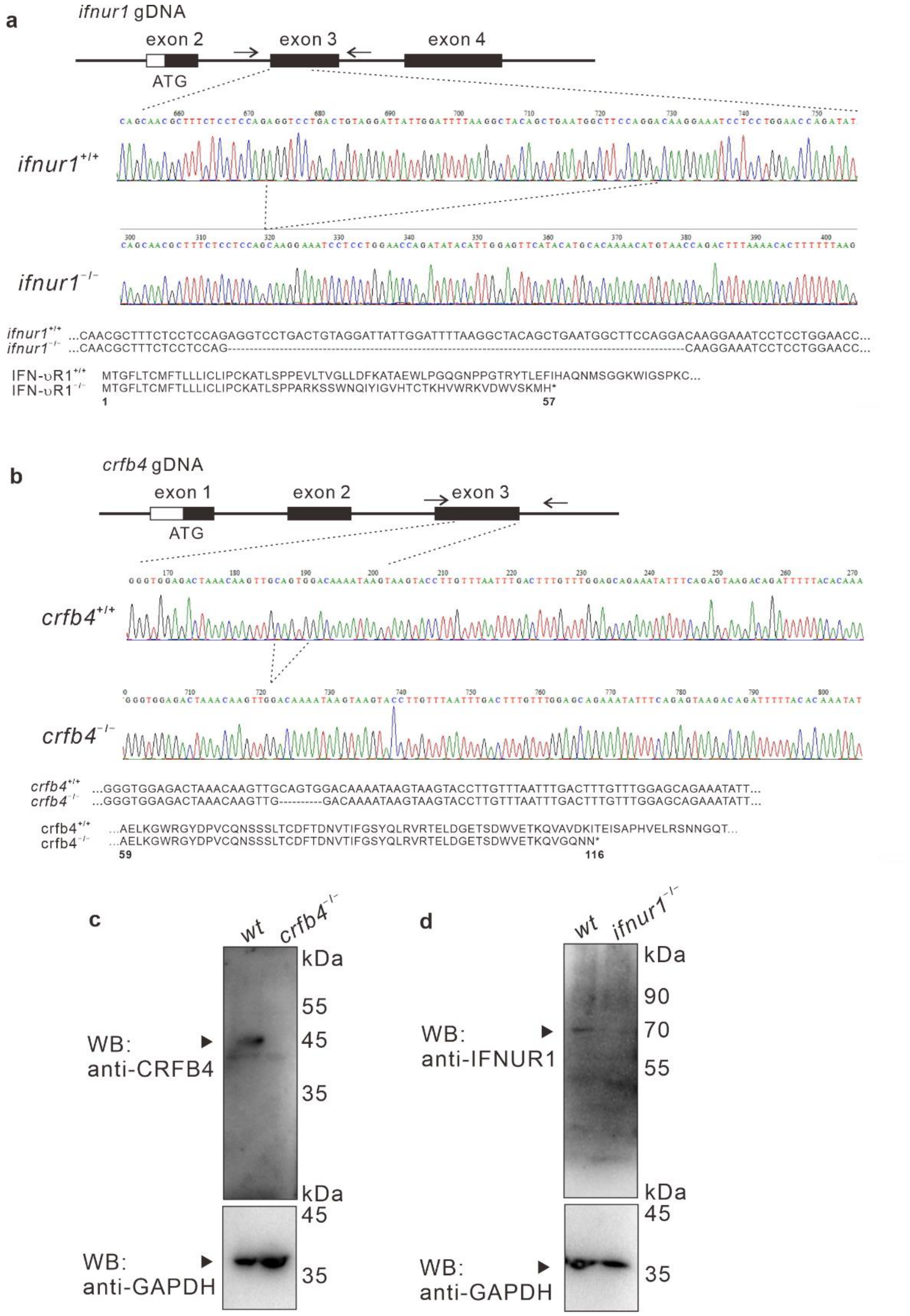

Supplementary Fig. 7. Generation of *ifnur1* and *crfb4* deficiency zebrafish. (a, b) Cas9/gRNA system specific to *ifnur1* (a) and *crfb4* (b) targets on Exon 3, and leads to 56 bp (5-AGGTCCTGACTGTAGGATTATTGGATTTTAAGGCTACAGCTGAATGGCTTCCAGGA-3) and 5 bp (5-CAGTG-3) deletion, respectively, which results in frameshift mutation and premature translation termination. (c, d) Detection of protein expression for CRFB4 (c) and IFNUR1 (d) in deficiency and WT zebrafish by Western blotting. GAPDH protein was detected as control. Data represent two independent experiments.

Supplementary Figure 8

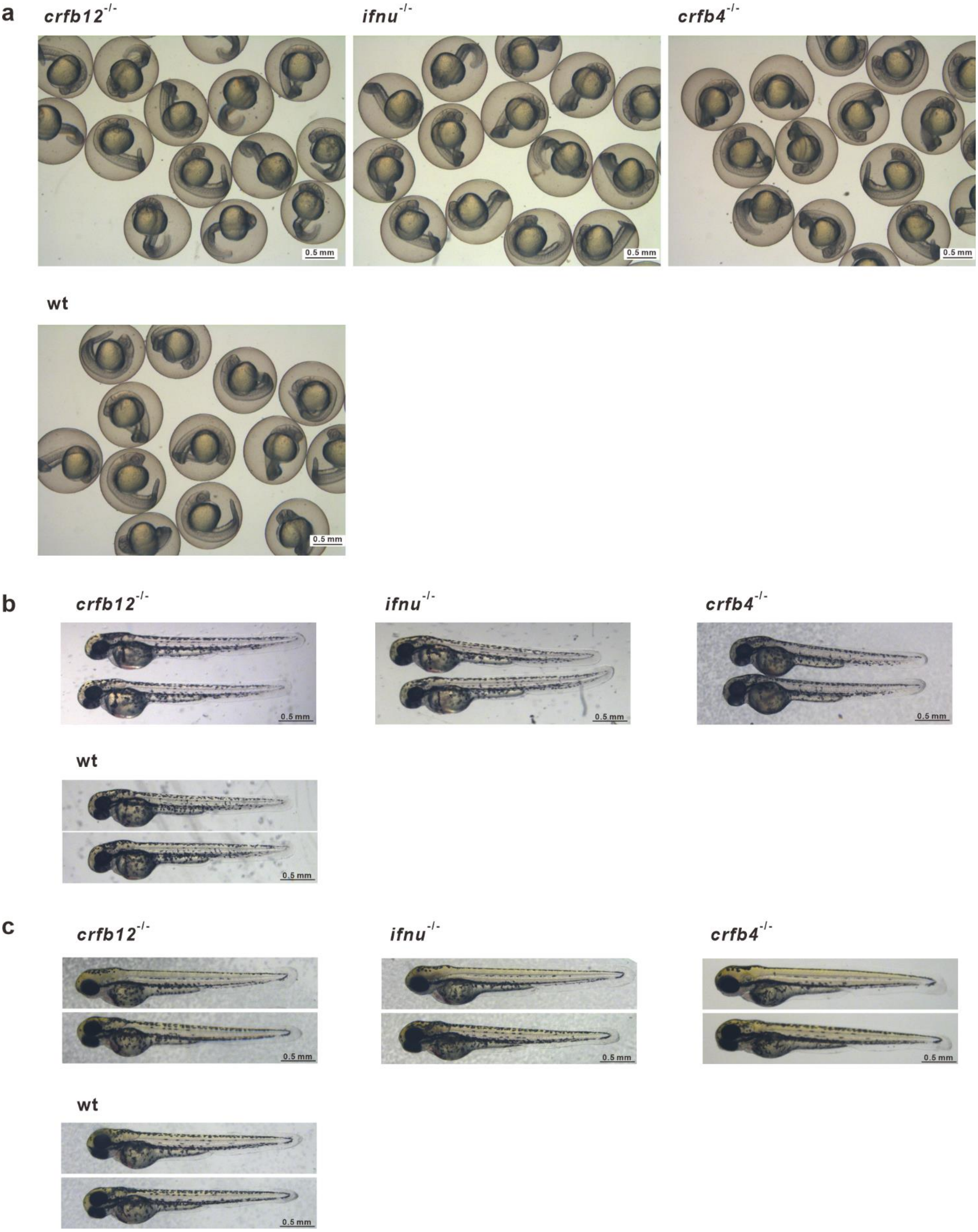

Supplementary Fig. 8. Observation of embryonic development phenotype for *ifnu*, *ifnur1* (*crfb12*) and *crfb4* knockout at 24 (a), 48 (b) and 72 (c) hpf.

Supplementary Figure 9

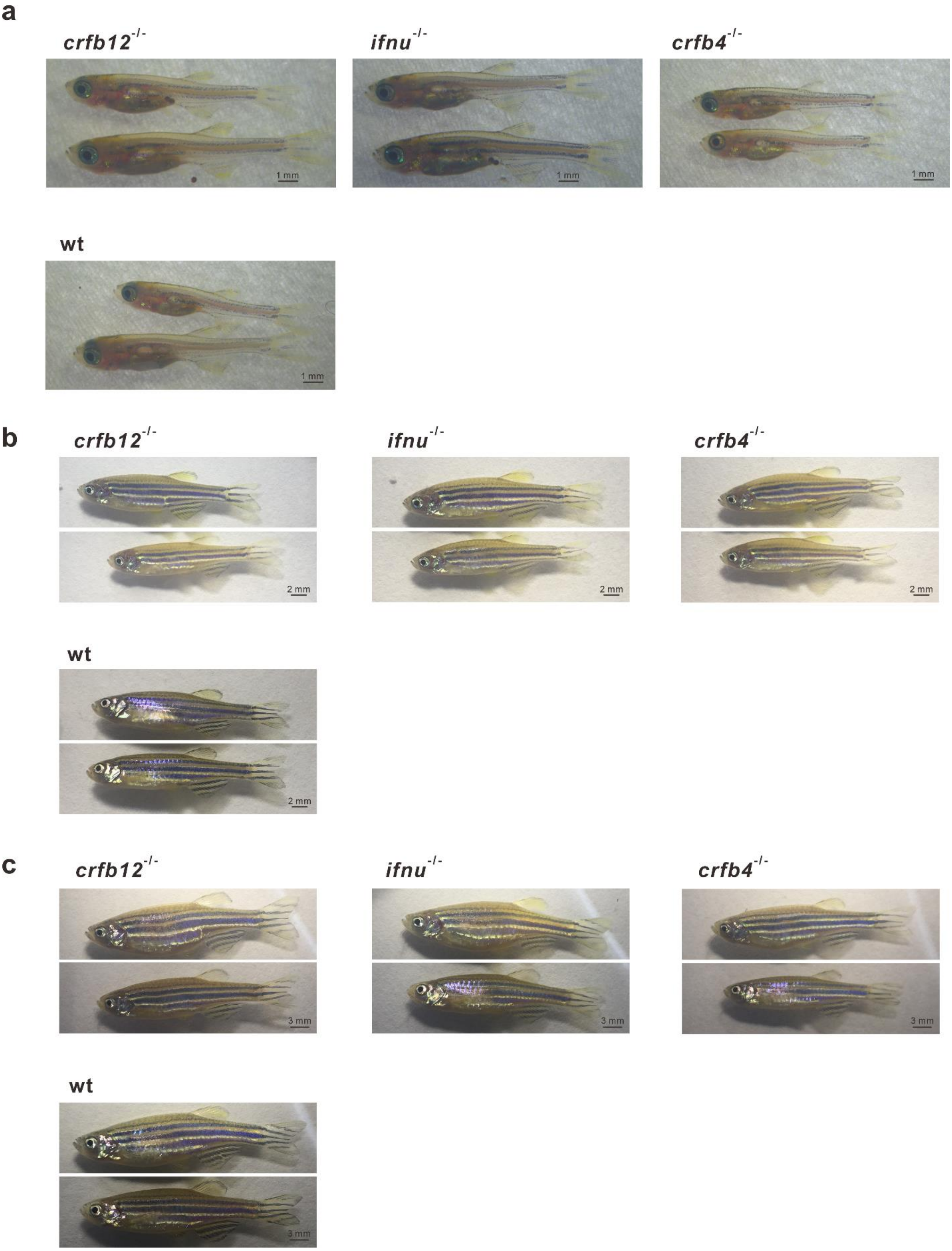

Supplementary Fig. 9. Observation of embryonic development phenotype for *ifnu*, *ifnur1* (*crfb12*) and *crfb4* knockout at 30 (a), 60 (b) and 90 (c) dpf.

Supplementary Figure 10

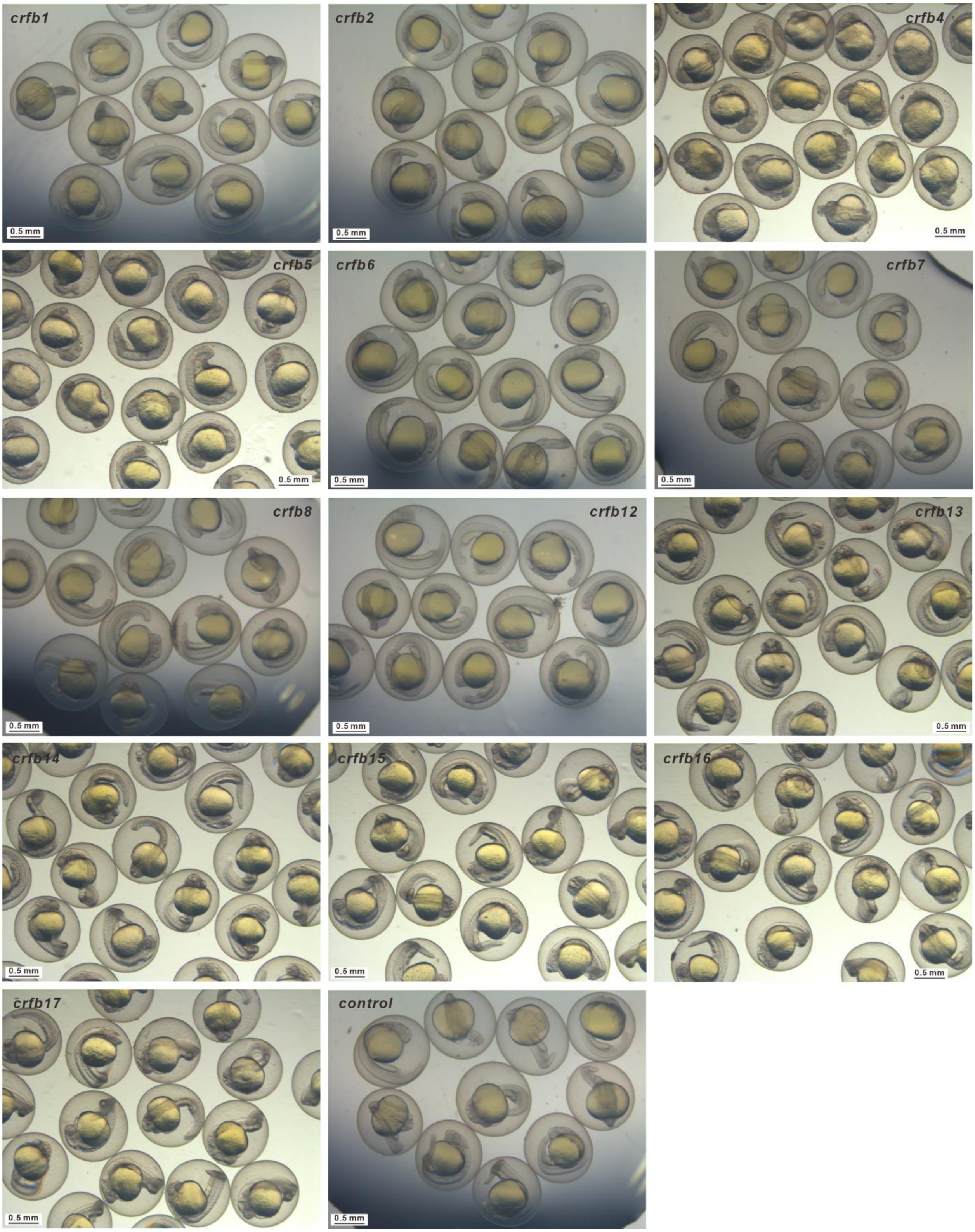

Supplementary Fig. 10. Observation of embryonic development phenotypes for the knockdown of CRFBs and control at 24 hpf. Different morpholinos for these CRFBs (1, 2, 4, 5, 6, 7, 8, 12, 13, 14, 15, 16 and 17) were injected separately into one-cell stage embryos, and 24 hours later embryos were collected for observation, respectively.

Supplementary Figure 11

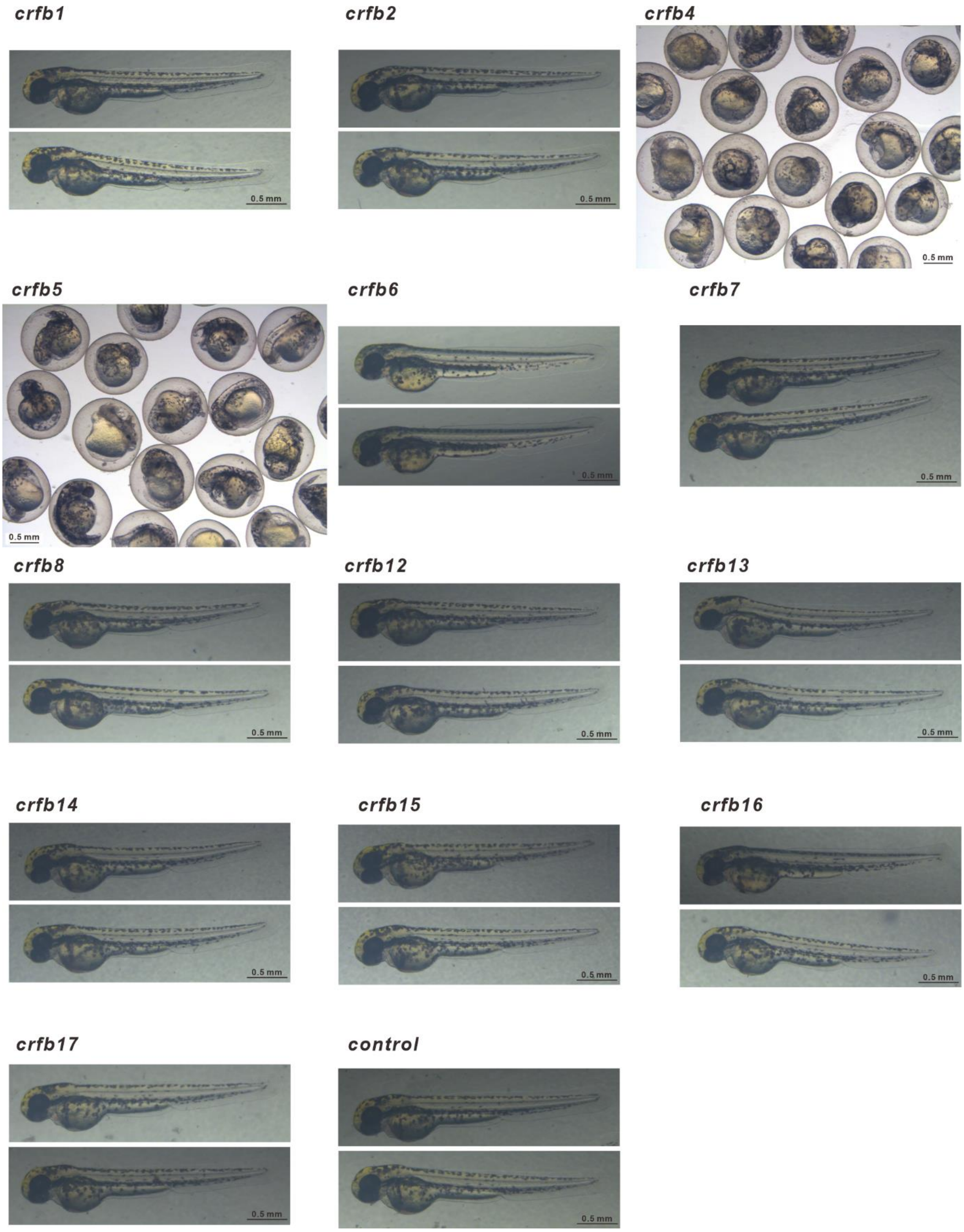

Supplementary Fig. 11. Observation of embryonic development phenotypes for the knockdown of CRFBs and control 48 hpf. Different morpholinos for these CRFBs (1, 2, 4, 5, 6, 7, 8, 12, 13, 14, 15, 16 and 17) were injected separately into one-cell stage embryos, and 48 hours later embryos were collected for observations, respectively.

Supplementary Figure 12

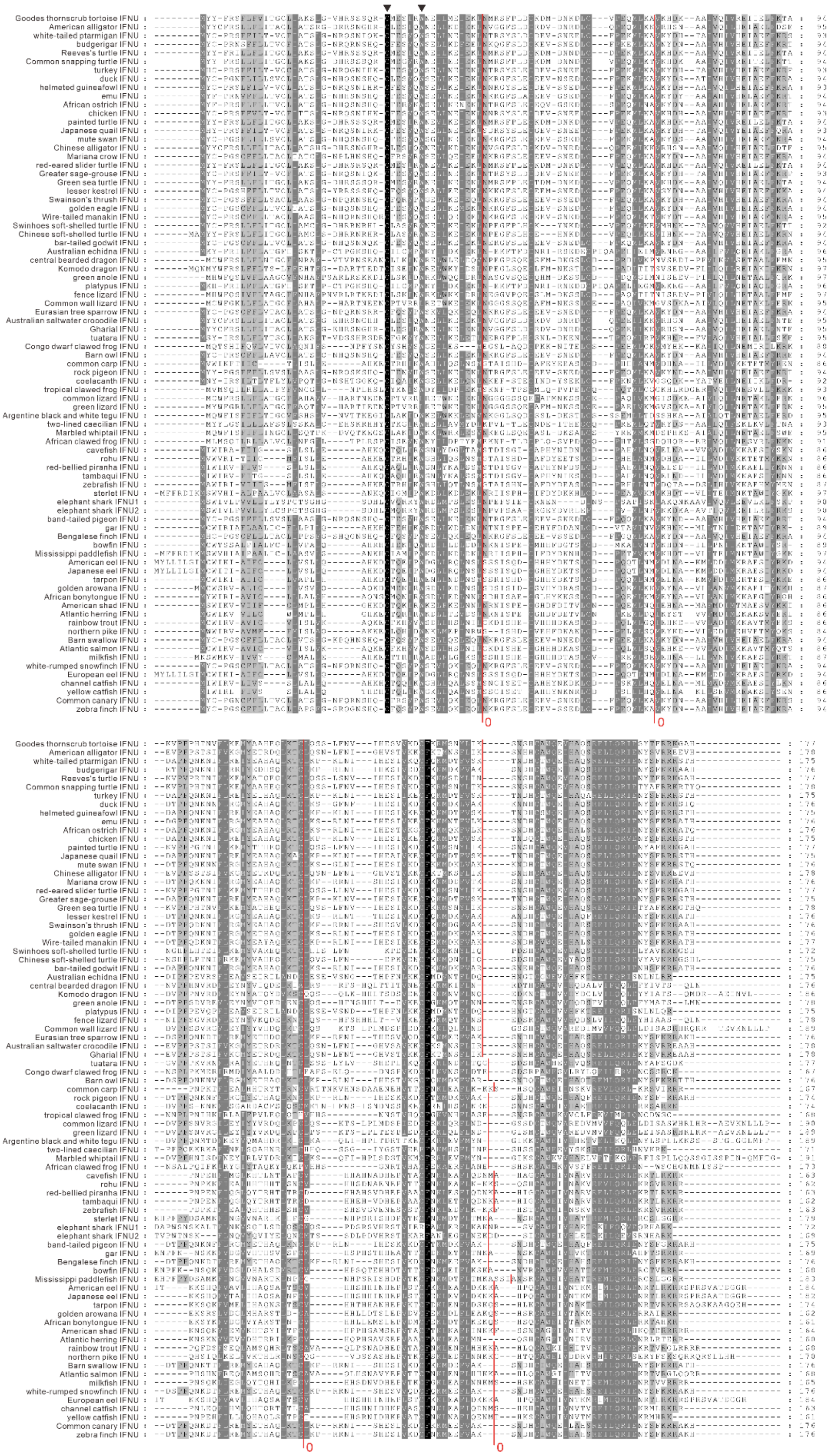

Supplementary Fig. 12. Sequence alignment of IFN-υ proteins in vertebrates. The conserved CXXXXX[W/L] sequences are marked in arrows. Identical and similar amino acids are indicated by shade with black and grey, respectively. The intron positions and their phases were indicated by red line.

Supplementary Figure 13

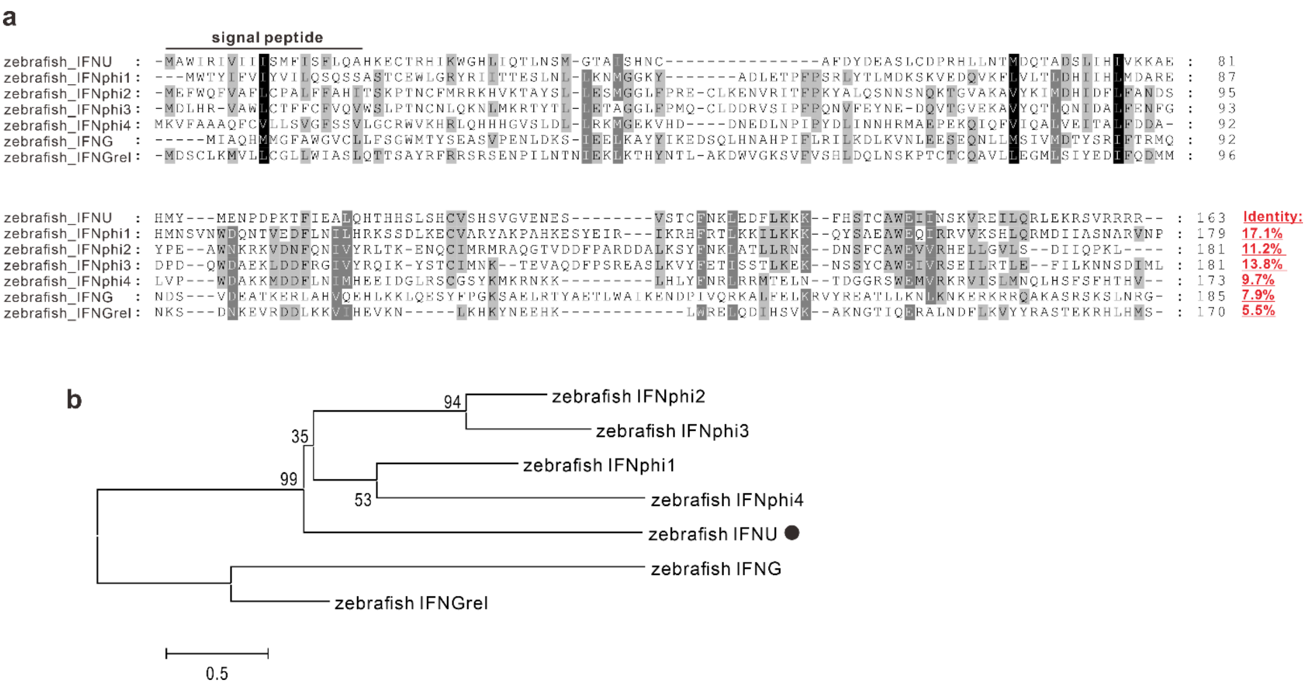

Supplementary Fig. 13. Sequence alignment (a) and phylogenetic analysis (b) of IFN genes in zebrafish. Identical and similar amino acids are indicated by shade with black and grey, respectively. The putative signal peptides are highlighted by solid lines above the alignment. Neighbor-joining (NJ) method was used to construct the phylogenetic tree.

Supplementary Figure 14

a

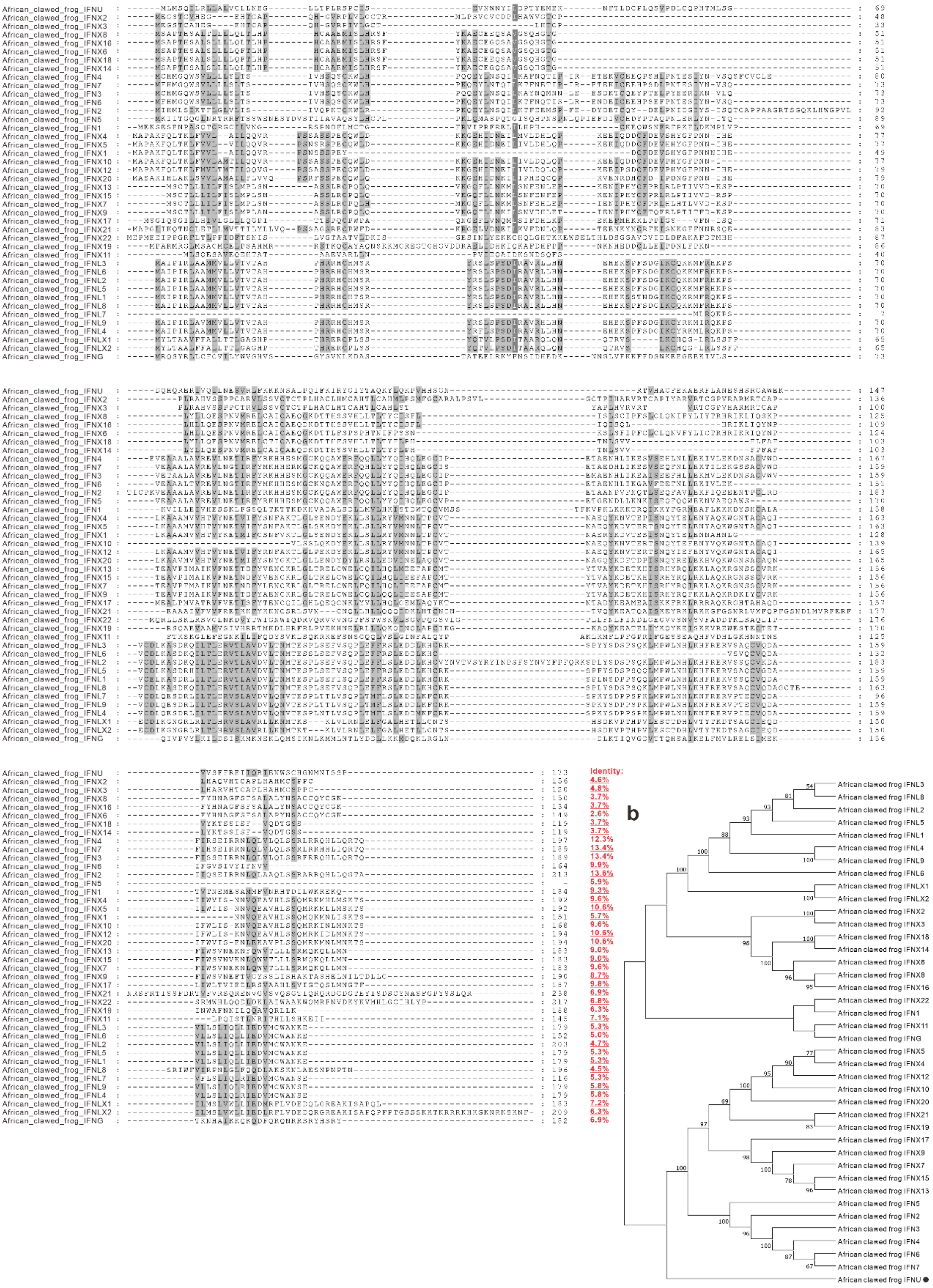

Supplementary Fig. 14. Sequence alignment (a) and phylogenetic analysis (b) of the IFN genes in clawed frog. Identical and similar amino acids are indicated by shade with black and grey, respectively. The putative signal peptides are highlighted by solid lines above the alignment. Neighbor-joining (NJ) method was used to construct the phylogenetic tree.

Supplementary Figure 15

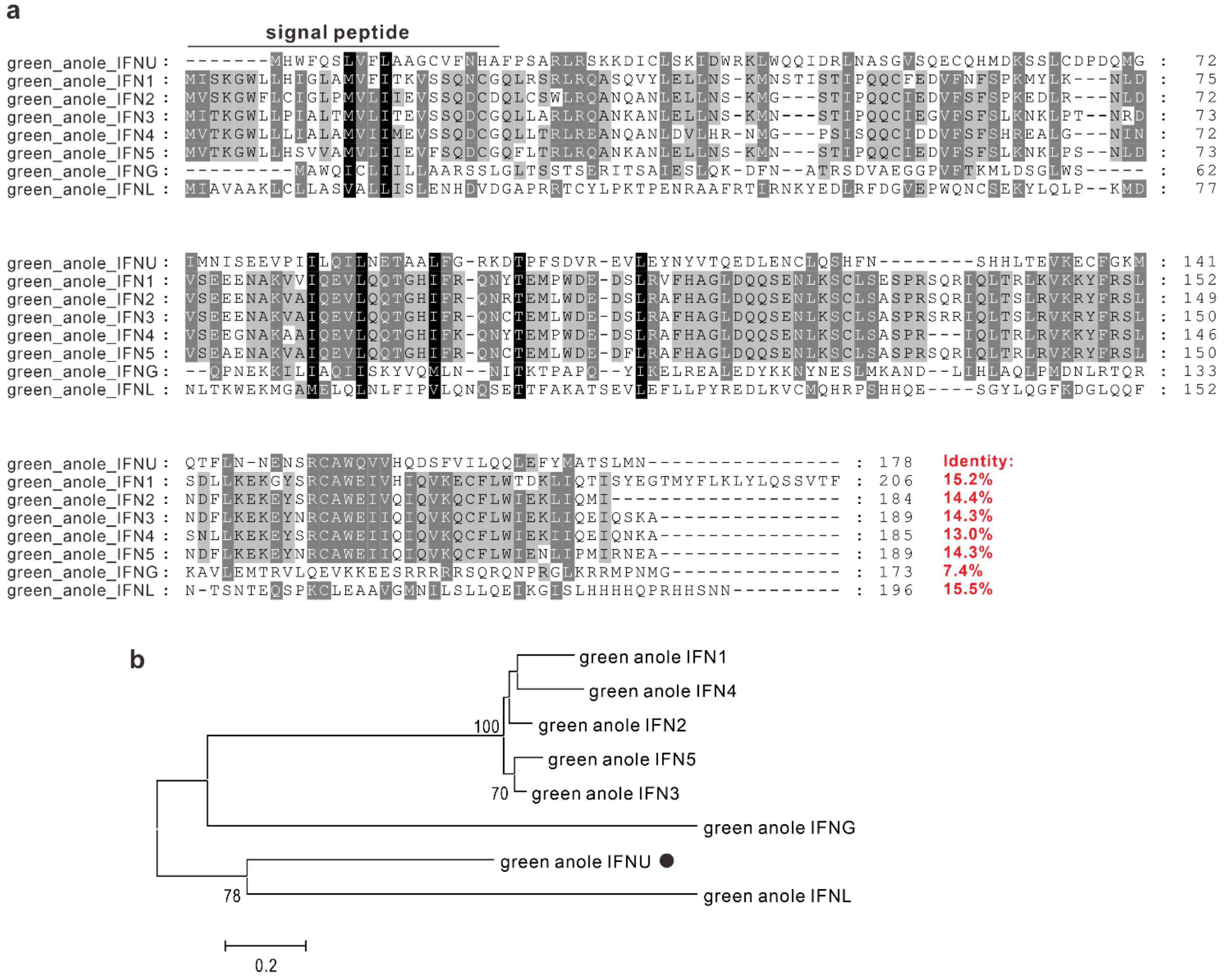

Supplementary Fig. 15. Sequence alignment (a) and phylogenetic analysis (b) of IFN genes in green anole. Identical and similar amino acids are indicated by shade with black and grey, respectively. The putative signal peptides are highlighted by solid lines above the alignment. Neighbor-joining (NJ) method was used to construct the phylogenetic tree.

Supplementary Figure 16

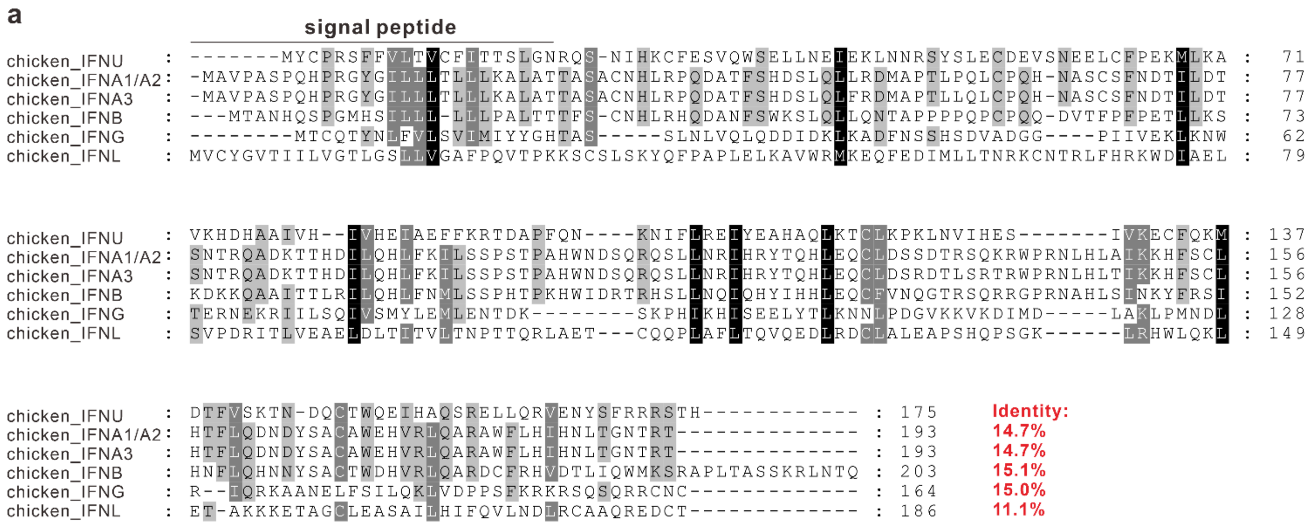

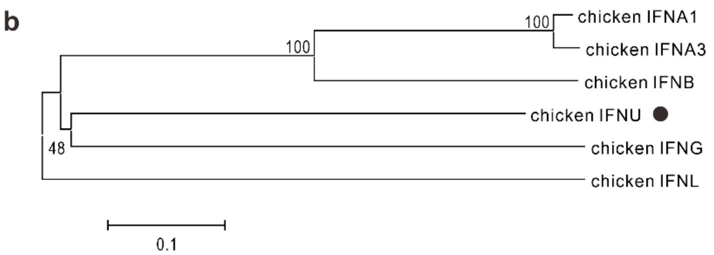

Supplementary Fig. 16. Sequence alignment (a) and phylogenetic analysis (b) of IFN genes in chicken. Identical and similar amino acids are indicated by shade with black and grey, respectively. The putative signal peptides are highlighted by solid lines above the alignment. Neighbor-joining (NJ) method was used to construct the phylogenetic tree.

Supplementary Figure 17

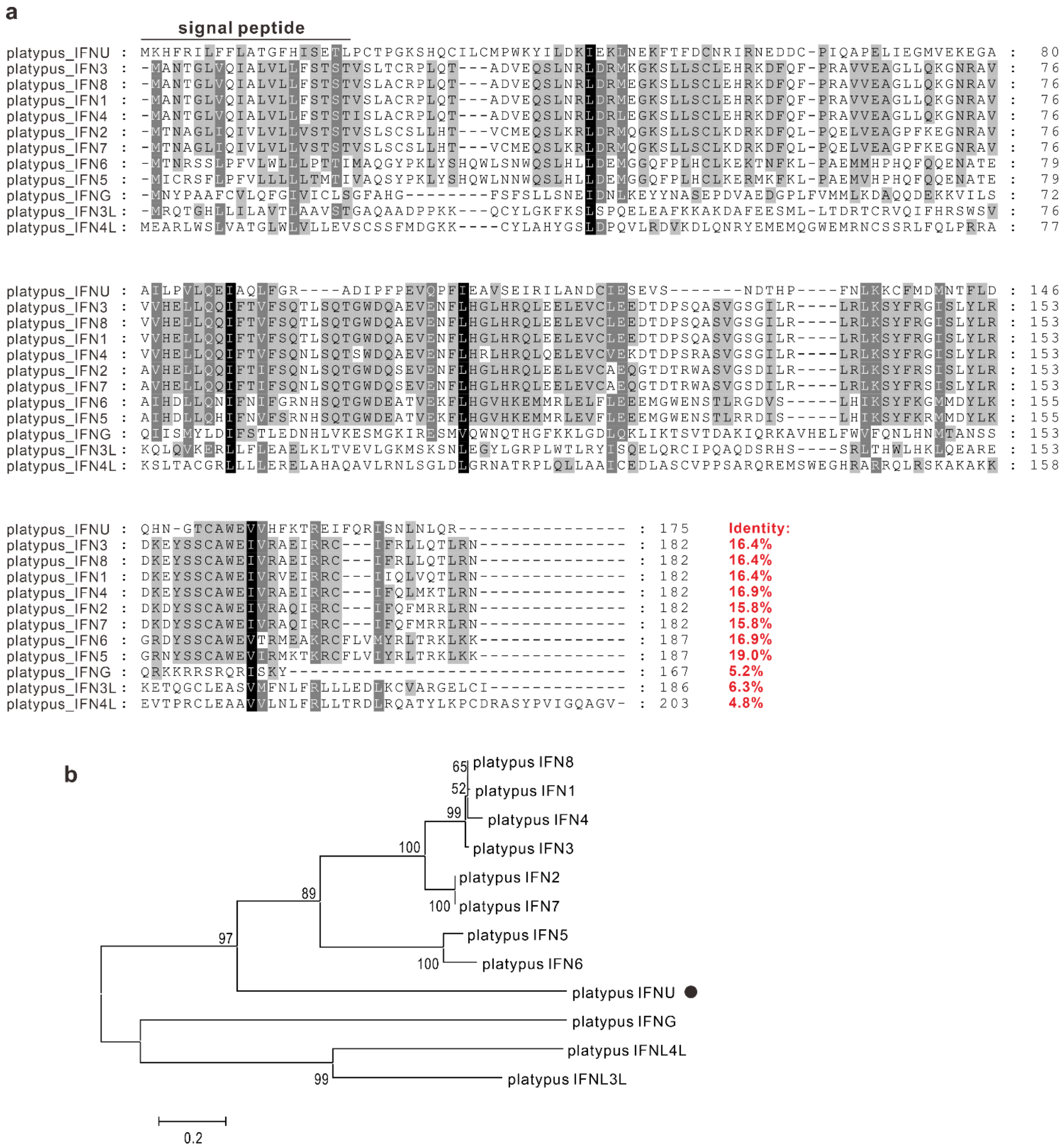

Supplementary Fig. 17. Sequence alignment (a) and phylogenetic analysis (b) of IFN genes in platypus. Identical and similar amino acids are indicated by shade with black and grey, respectively. The putative signal peptides are highlighted by solid lines above the alignment. Neighbor-joining (NJ) method was used to construct the phylogenetic tree.

Supplementary Figure 18

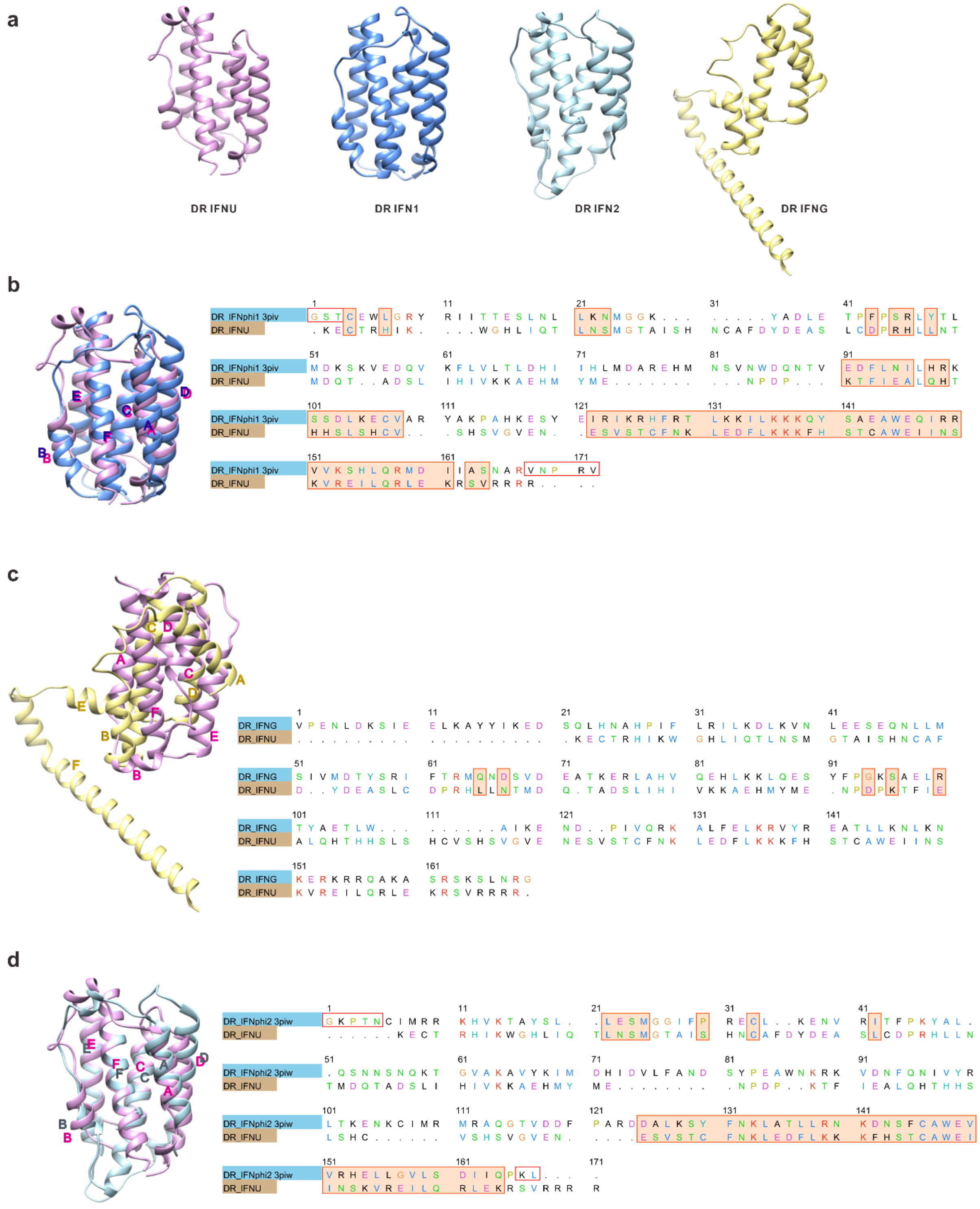

Supplementary Fig. 18. Structural comparison of IFNs in zebrafish. (a) The structures of type I (IFN-φ1/IFN1, 3piv and IFN-φ2/IFN2, 3piw) and putative structures of type II (IFNG) and IFNU. Comparison of IFNU to IFN-φ1 (b), IFNG (c) and IFN-φ2 (d). Putative helices of IFNU were indicated by A to E. The overlaid regions on structure were highlighted by orange box in the alignments.

Supplementary Figure 19

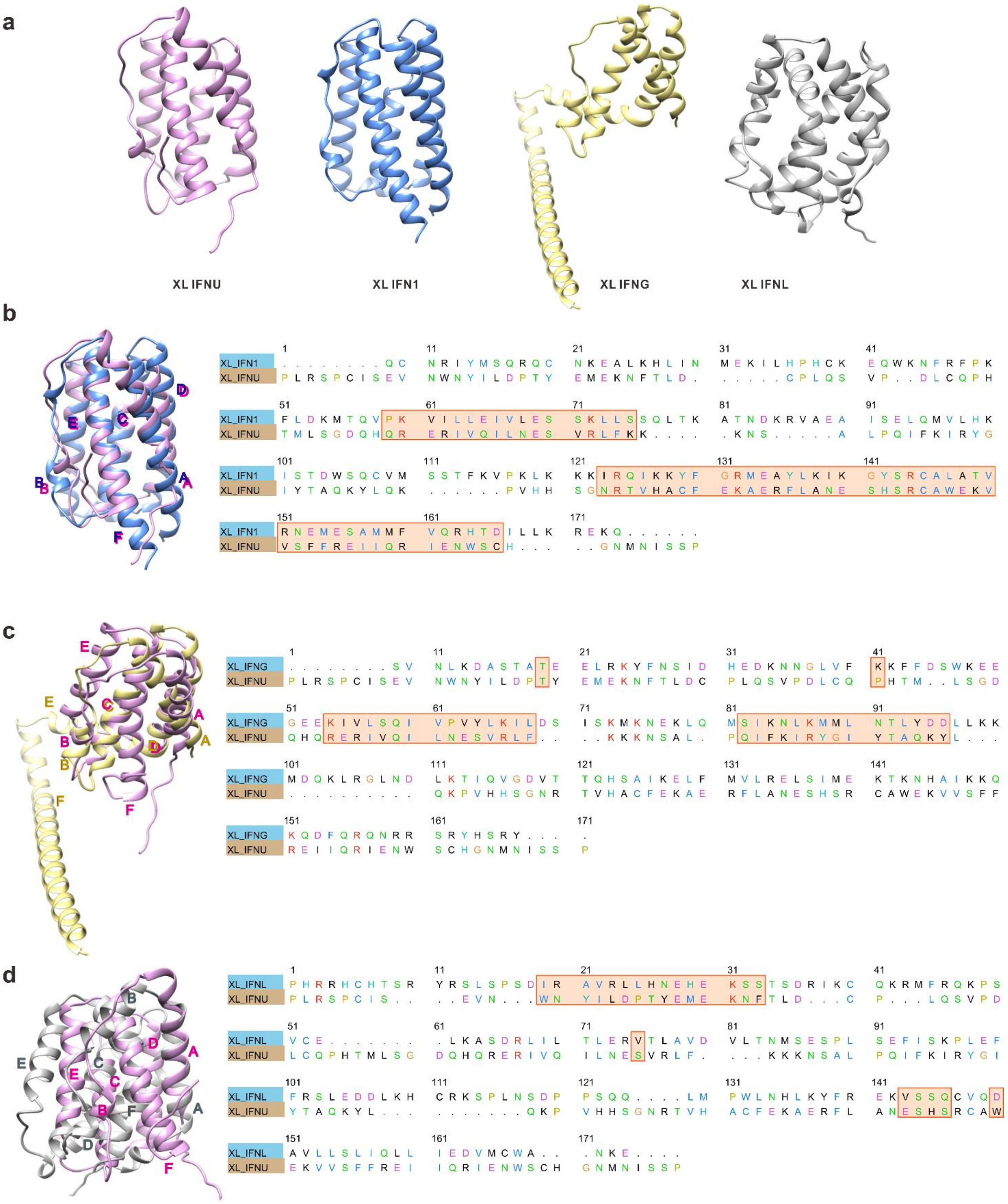

Supplementary Fig. 19. Structural comparison of IFNs in clawed frog. (a) The putative structures of type I (IFN1), type II (IFNG), type III (IFNL) and IFNU. Comparison of IFNU to IFN1 (b), IFNG (c) and IFNL (d). Putative helices of IFNU were indicated by A to E. The overlaid regions on structure were highlighted by orange box in the alignments.

Supplementary Figure 20

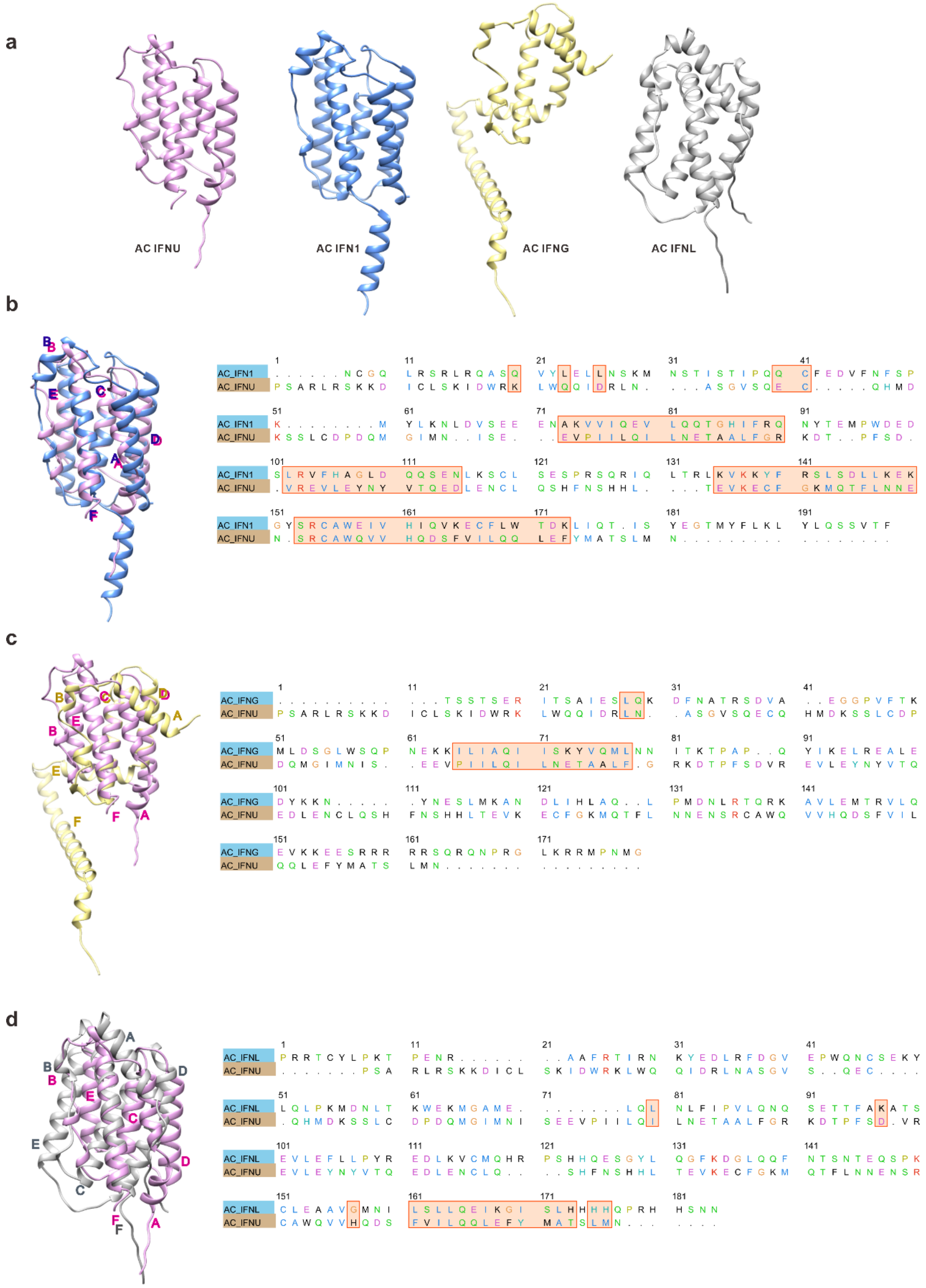

Supplementary Fig. 20. Structural comparison of IFNs in green anole. (a) The putative structures of type I (IFN1), type II (IFNG), type III (IFNL) and IFNU. Comparison of IFNU to IFN1 (b), IFNG (c) and IFNL (d). Putative helices of IFNU were indicated by A to E. The overlaid regions on structure were highlighted by orange box in the alignments.

Supplementary Figure 21

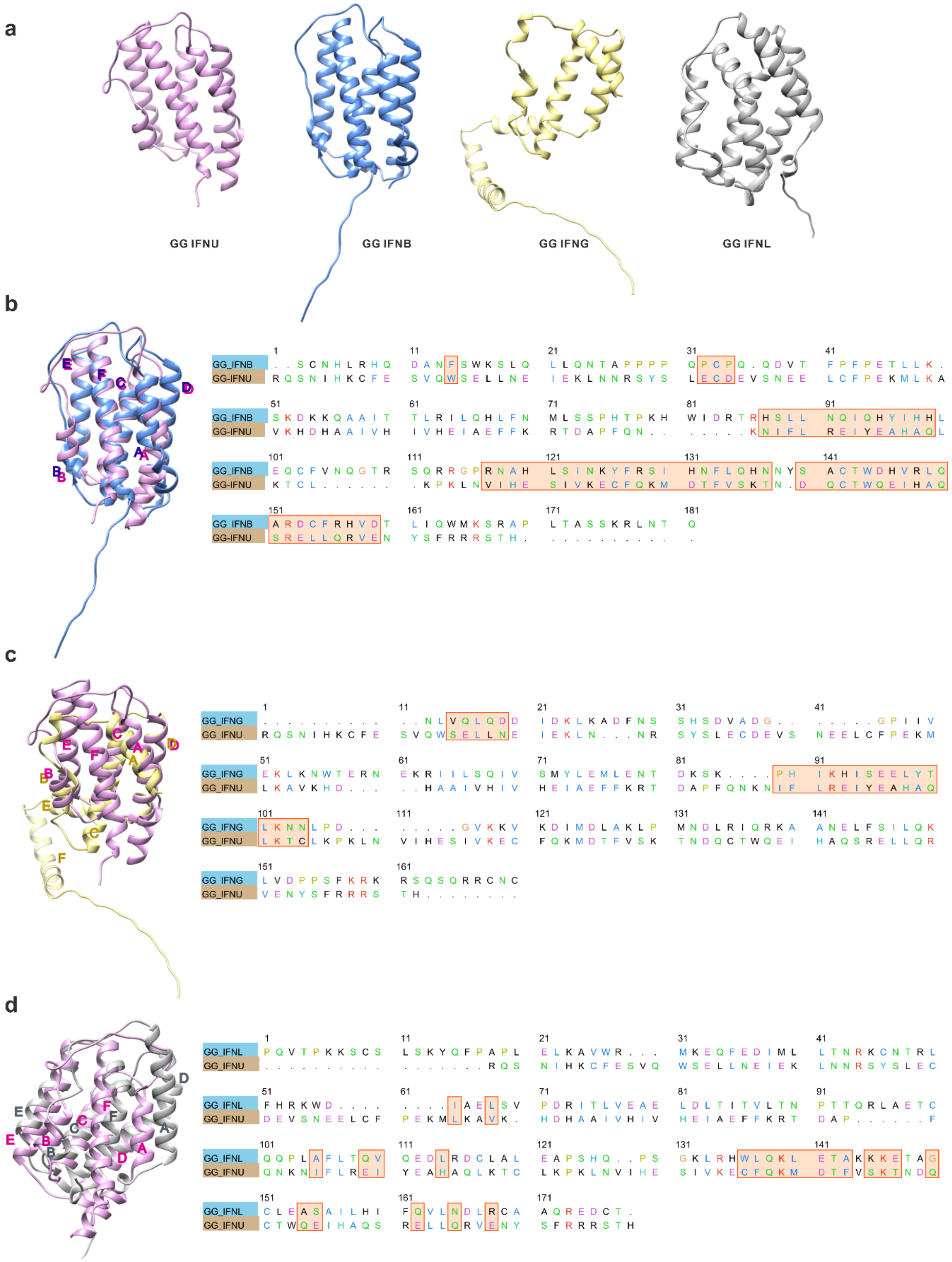

Supplementary Fig. 21. Structural comparison of IFNs in chicken. (a) The putative structures of type I (IFNB), type II (IFNG), type III (IFNL) and IFNU. Comparison of IFNU to IFNB (b), IFNG (c) and IFNL (d). Putative helices of IFNU were indicated by A to E. The overlaid regions on structure were highlighted by orange box in the alignments.

Supplementary Figure 22

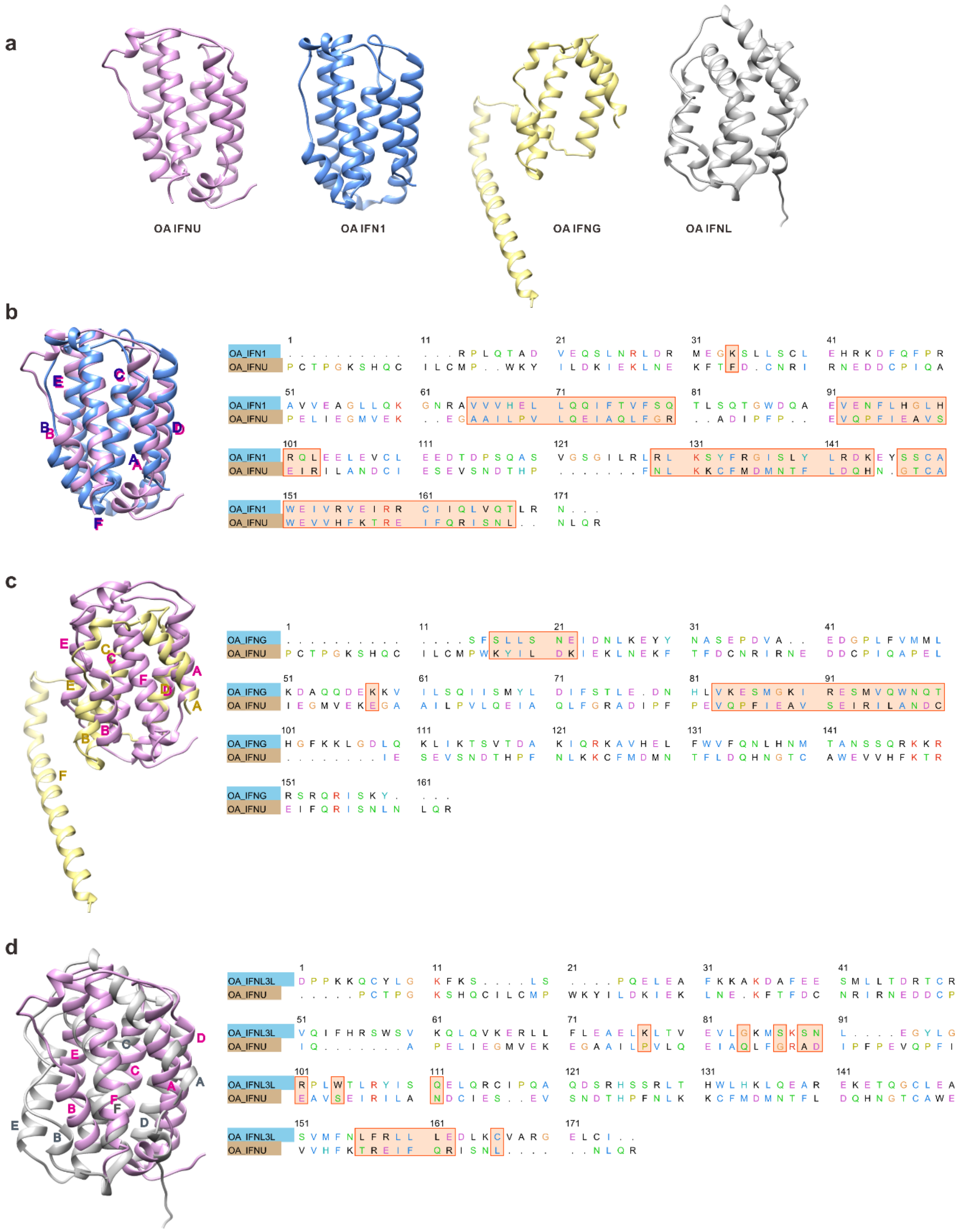

Supplementary Fig. 22. Structural comparison of IFNs in platypus. (a) The putative structures of type I (IFN1), type II (IFNG), type III (IFNL3L) and IFNU. Comparison of IFNU to IFN1 (b), IFNG (c) and IFNL3L (d). Putative helices of IFNU were indicated by A to E. The overlaid regions on structure were highlighted by orange box in the alignments.

Supplementary Figure 23

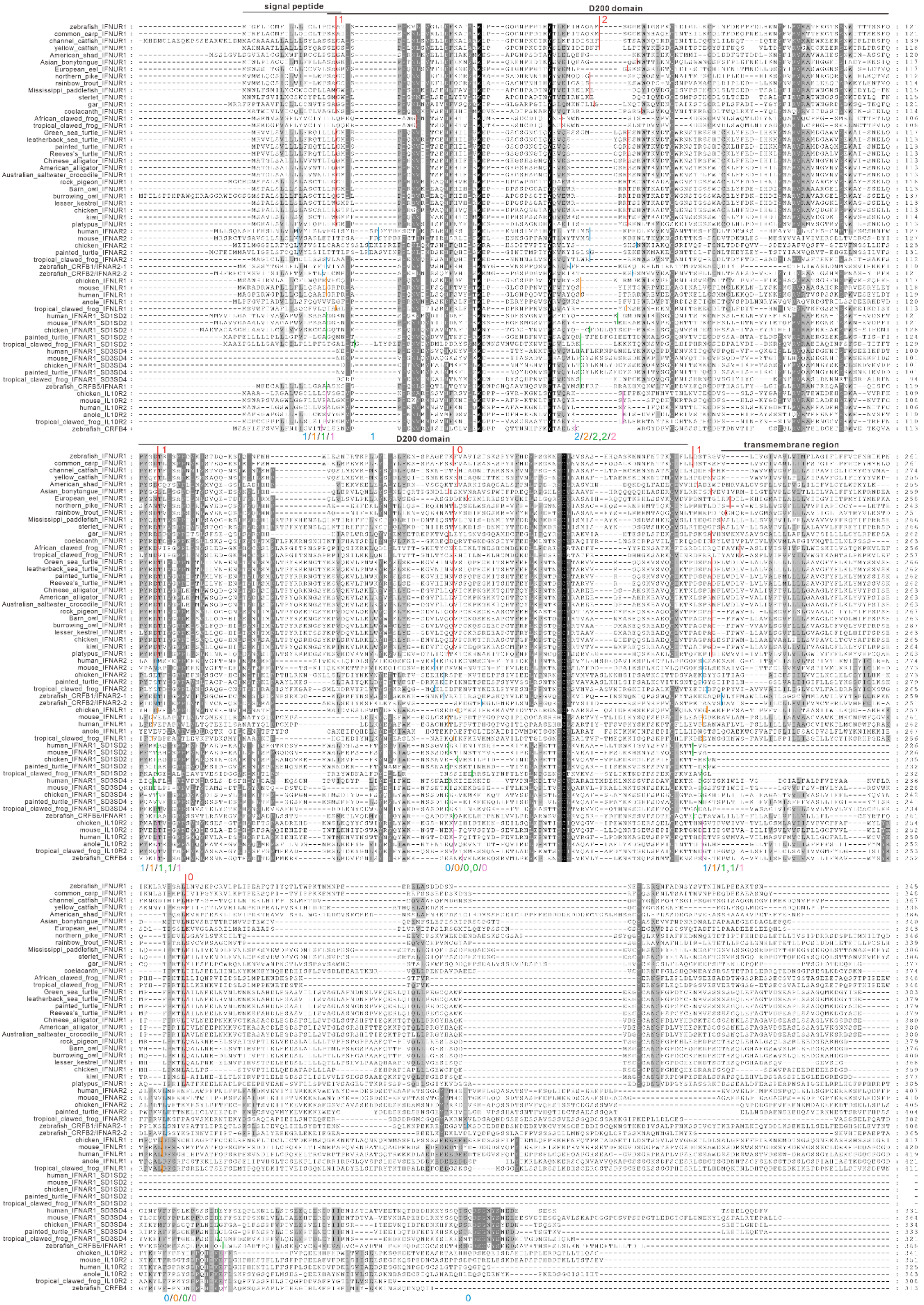

Supplementary Figure 23 – continued

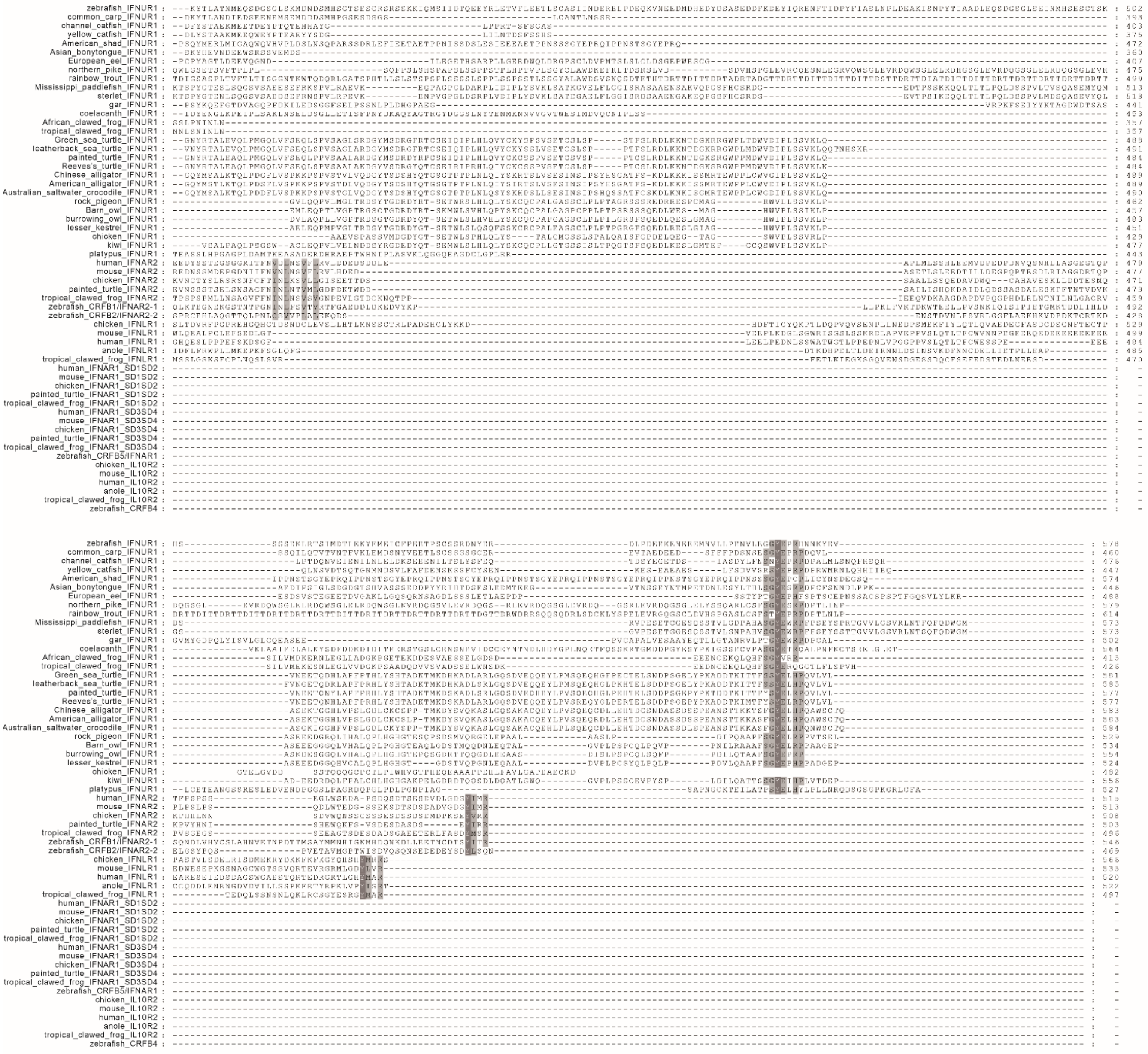

Supplementary Fig. 23. Sequence alignment of IFN receptor proteins in vertebrates. Identical and similar amino acids are indicated by shade with black and grey, respectively. The putative signal peptide, D200 domain and transmembrane region are highlighted by solid lines above the alignment. The intron positions and phases of receptor genes were indicated by red (IFNUR1), blue (IFNAR2), yellow (IFNLR1), green (IFNAR1) and pink (IL10R2) lines, respectively.

Supplementary Figure 24

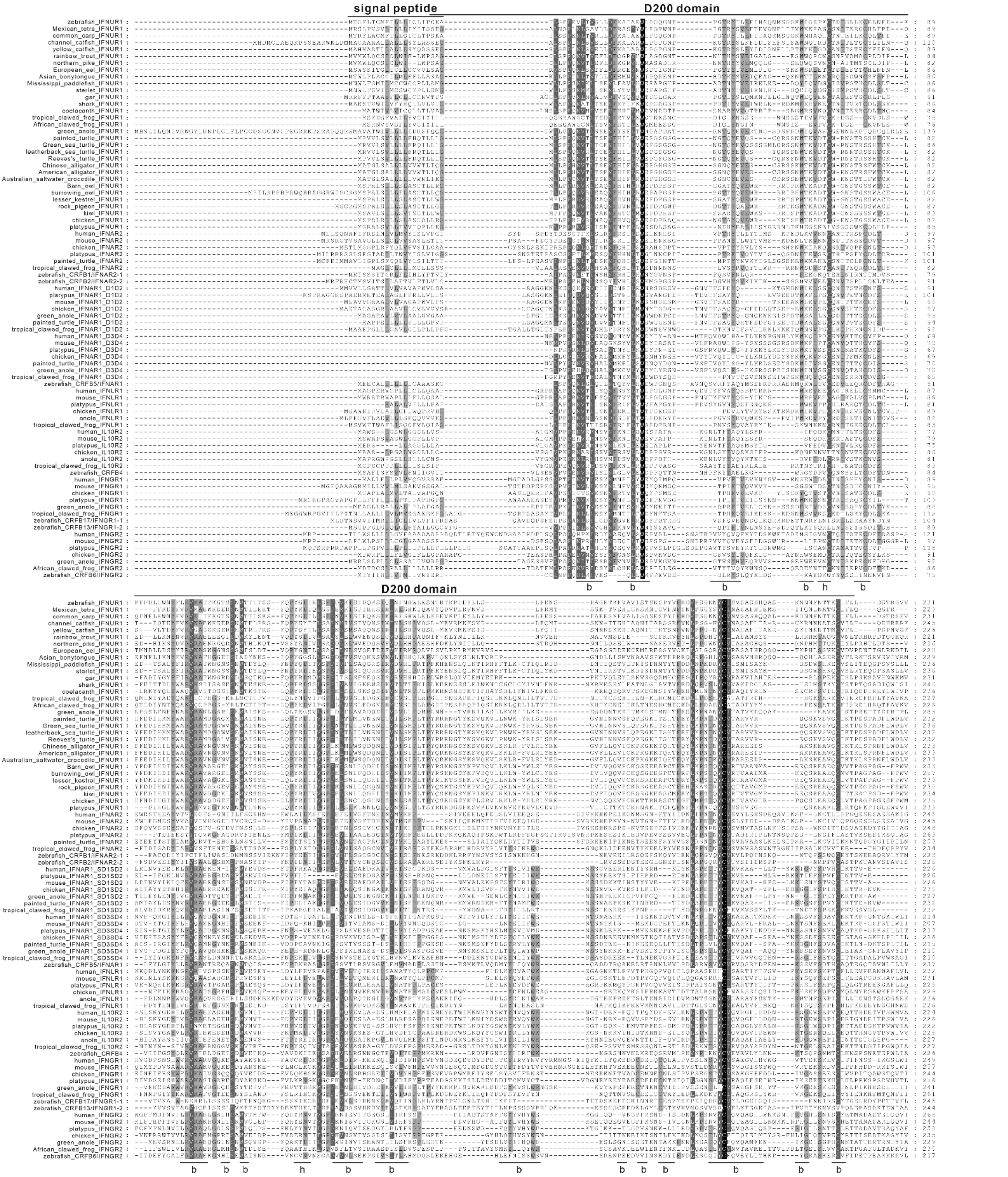

Supplementary Fig. 24. Sequence alignment of extracellular regions in IFN receptor proteins in vertebrates. Identical and similar amino acids are indicated by shade with black and grey, respectively. The putative signal peptide and D200 domain are highlighted by solid lines above the alignment. The helices and beta-sheet related structures of IFNLR1 are indicated by underline with “h” and “b”, respectively.

Supplementary Figure 25

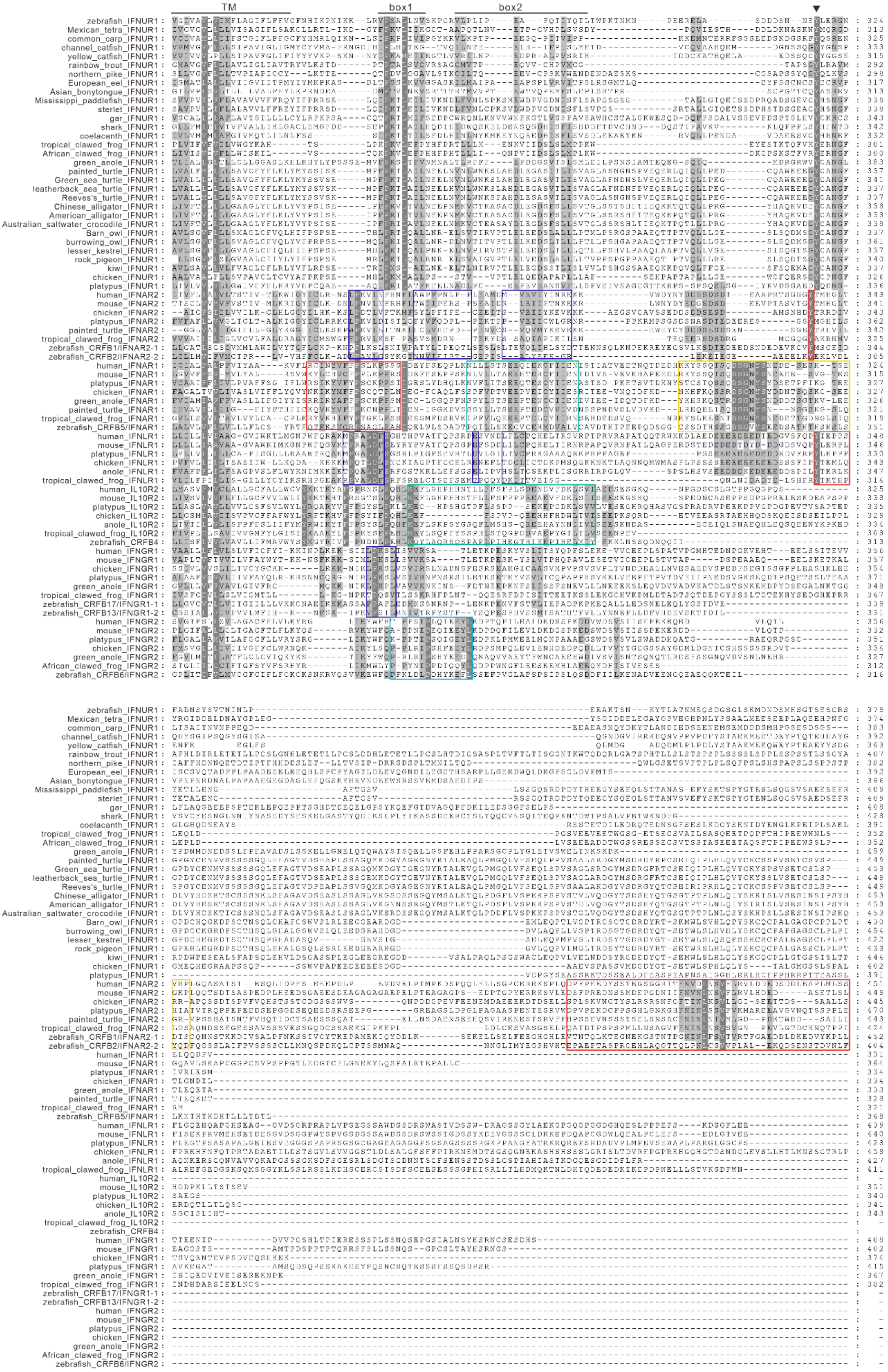

Supplementary Figure 25 – continued

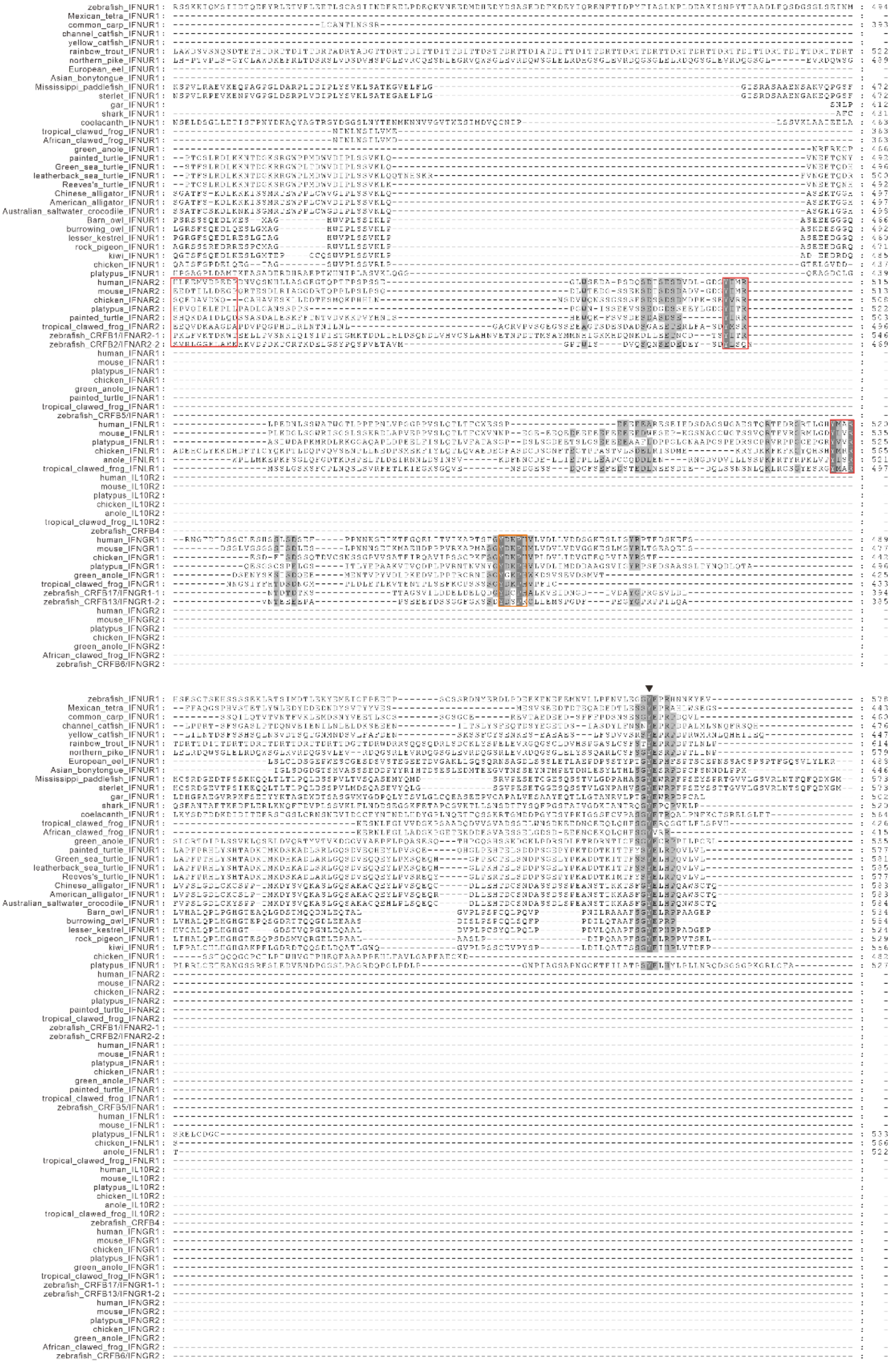

Supplementary Fig. 25. Sequence alignment of intracellular regions in IFN receptor proteins in vertebrates. Identical and similar amino acids are indicated by shade with black and grey, respectively. The conserved peptide motif (PXXL) and a hydrophobic residues-rich sequence of IFNUR1 are indicated by 'box1' and 'box2' above the alignment, respectively. The conserved tyrosine of IFNUR1 is marked by arrows. The known docking sites for JAK1, JAK2, TYK2, STAT1, STAT2 and STAT3 are indicated by dark blue, light blue, green, orange, red and yellow box, respectively.

Supplementary Figure 26

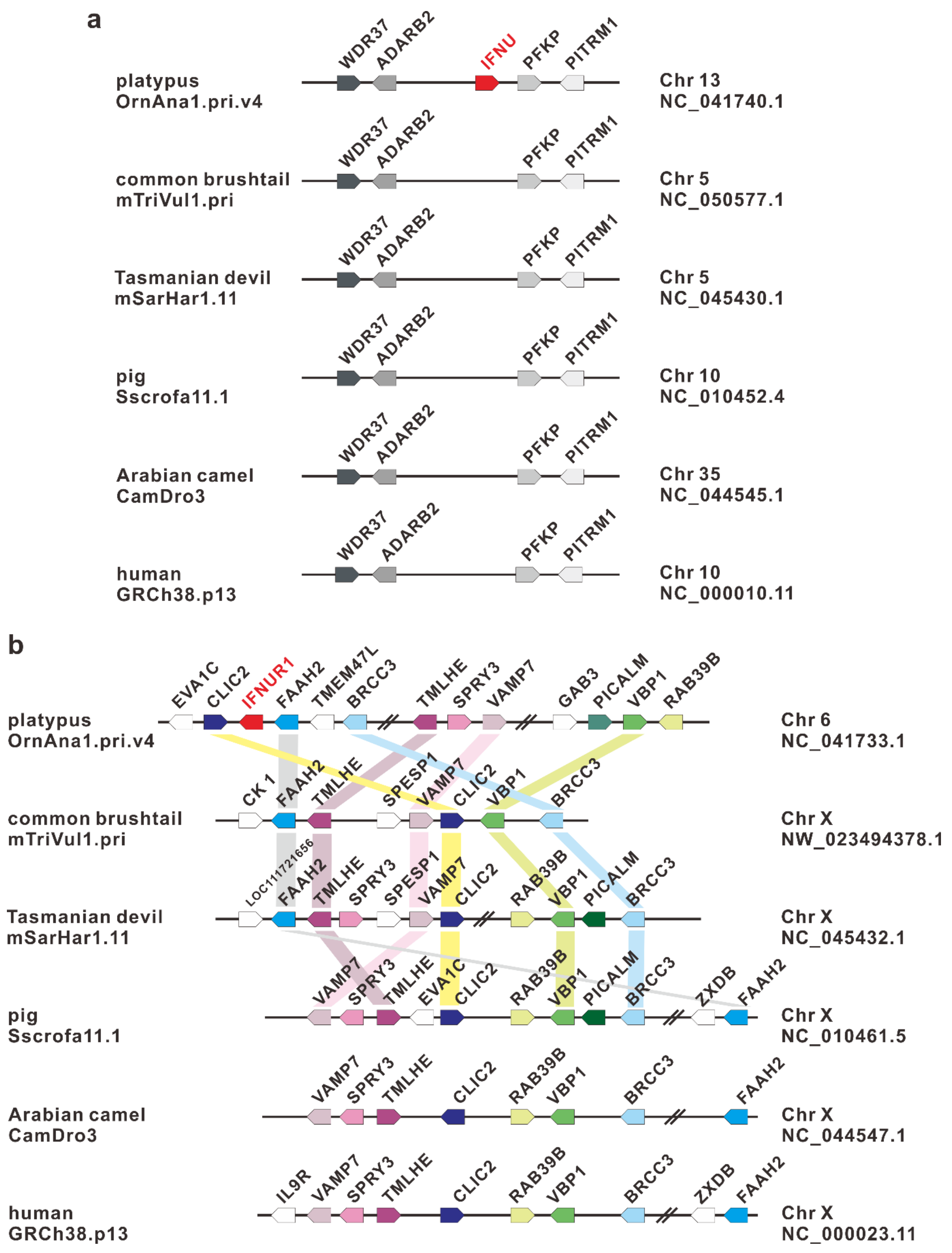

Supplementary Fig. 26. Collinearity analysis of *ADARB2-PFKP* locus (a) and *CLIC2-BRCC3* locus (b) in mammals. All genes are indicated with arrow symbols which point to the transcription direction. *IFNU* and *IFNUR1* are dyed in red. Gene name abbreviation: WDR37, WD repeat domain 37; ADARB2, adenosine deaminase RNA specific B2 (inactive); PFKP, phosphofructokinase, platelet; PITRM1, pitrilysin metallopeptidase 1; FAAH2, fatty acid amide hydrolase 2; TMLHE, trimethyllysine hydroxylase, epsilon; VAMP7, vesicle associated membrane protein 7; VBP1, VHL binding protein 1; SPRY3, sprouty RTK signaling antagonist 3; RAB39B, RAB39B, member RAS oncogene family; BRCC3, BRCA1/BRCA2-containing complex subunit 3; PICALM, phosphatidylinositol binding clathrin assembly protein; ZXDB, zinc finger X-linked duplicated B.

Supplementary Figure 27

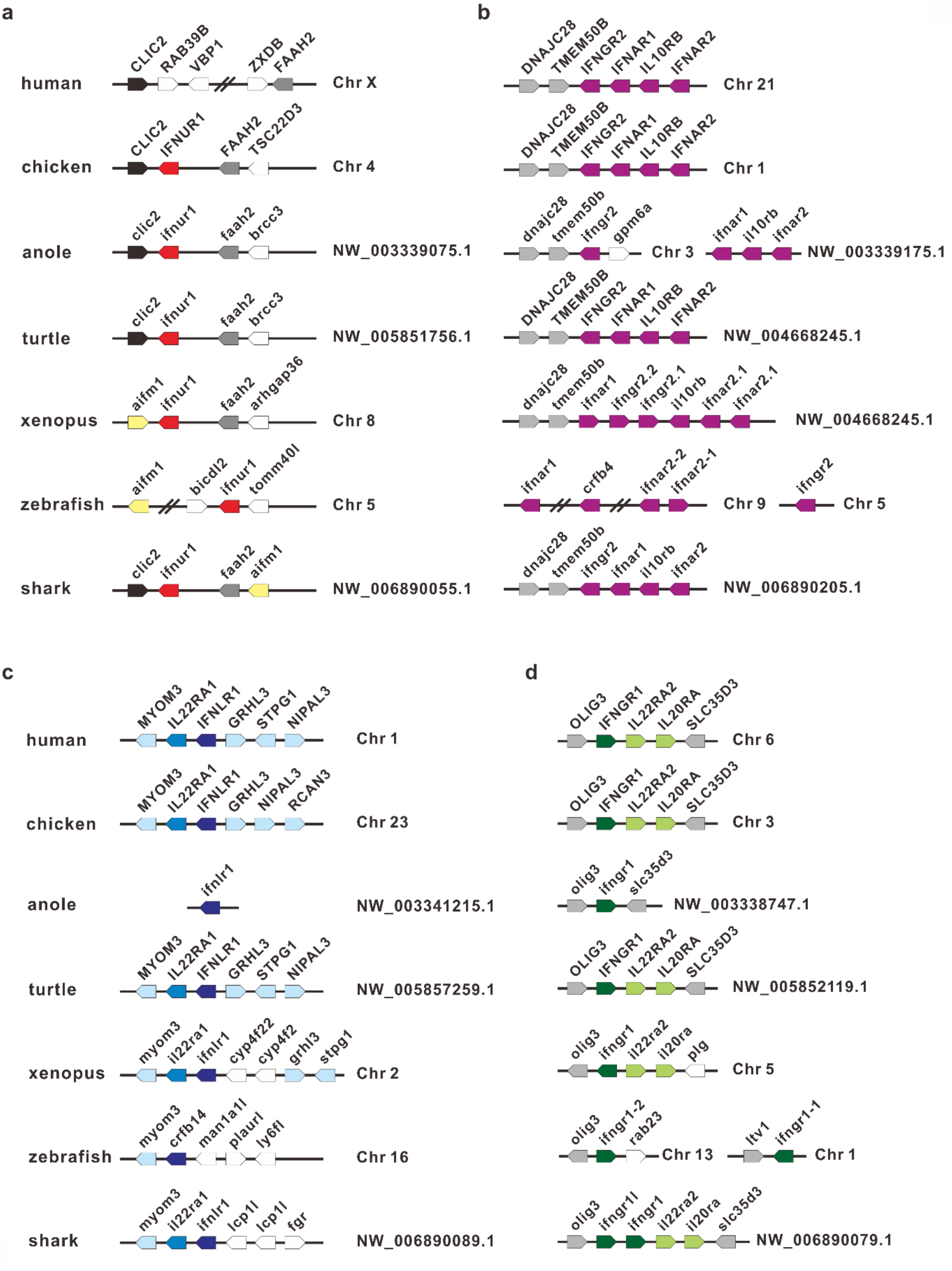

Supplementary Fig. 27. Gene synteny of *IFNUR1* (a) and other IFN receptor gene (b-d) loci in vertebrates. All genes are indicated with arrow symbols which point to the transcription direction. *IFNUR1* are dyed in red, and the conserved and non-conserved neighbor genes are illustrated in grey and white, respectively. Gene name abbreviation: AIFM1, apoptosis inducing factor mitochondria associated 1; DNAJC28, DnaJ heat shock protein family (Hsp40) member C28; TMEM50B, transmembrane protein 50B; MYOM3, myomesin 3; GRHL3, grainyhead like transcription factor 3; STPG1, sperm tail PG-rich repeat containing 1; NIPAL3, NIPA like domain containing 3; RCAN3, RCAN family member 3; OLIG3, oligodendrocyte transcription factor 3; SLC35D3, solute carrier family 35 member D3.

Supplementary Figure 28

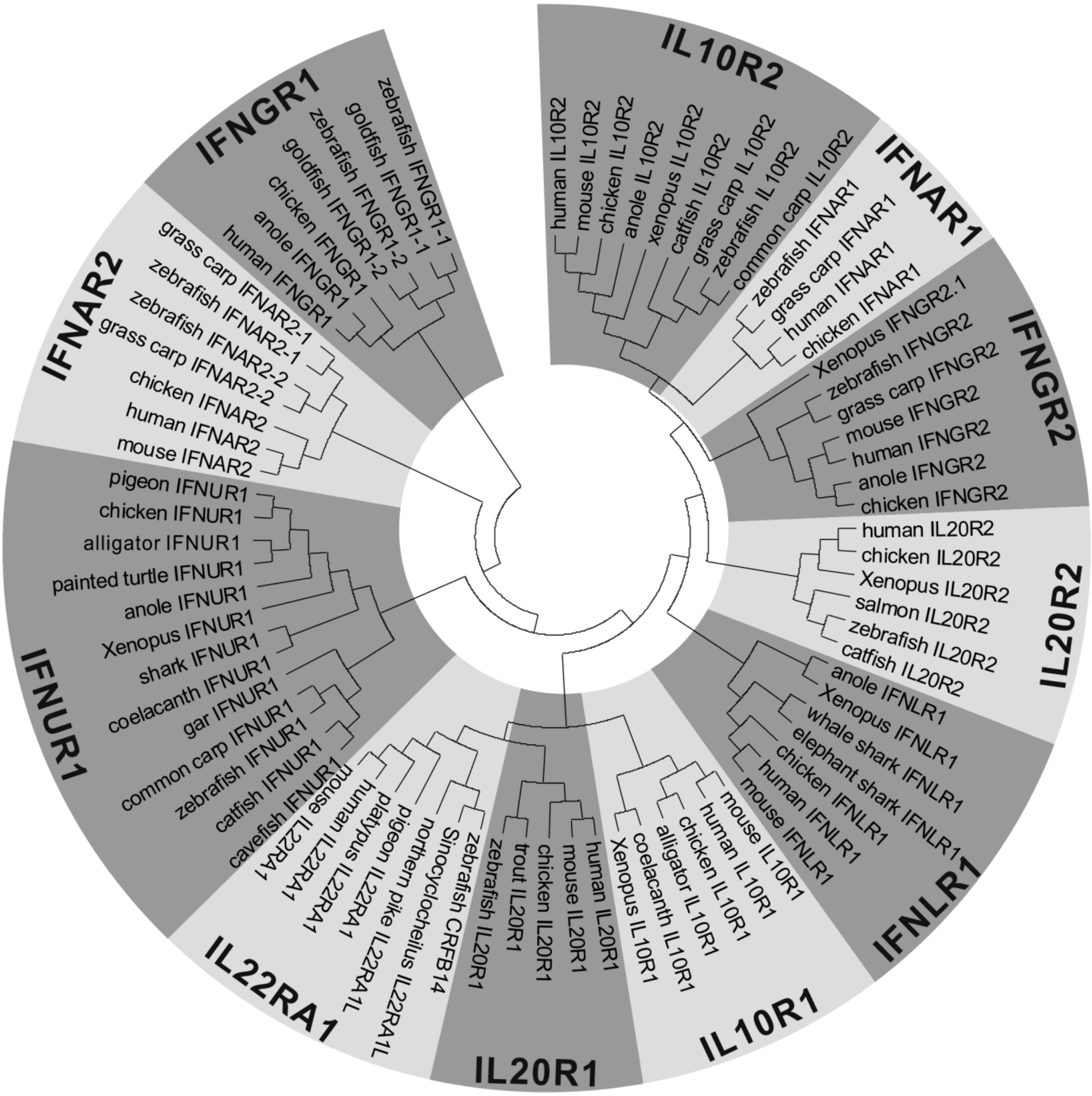

Supplementary Fig. 28. Phylogenetic analysis of IFNUR1 in vertebrates. Protein sequences from class II cytokine receptors in vertebrates were used to construct the neighbor-joining (NJ) tree.

Supplementary Figure 29

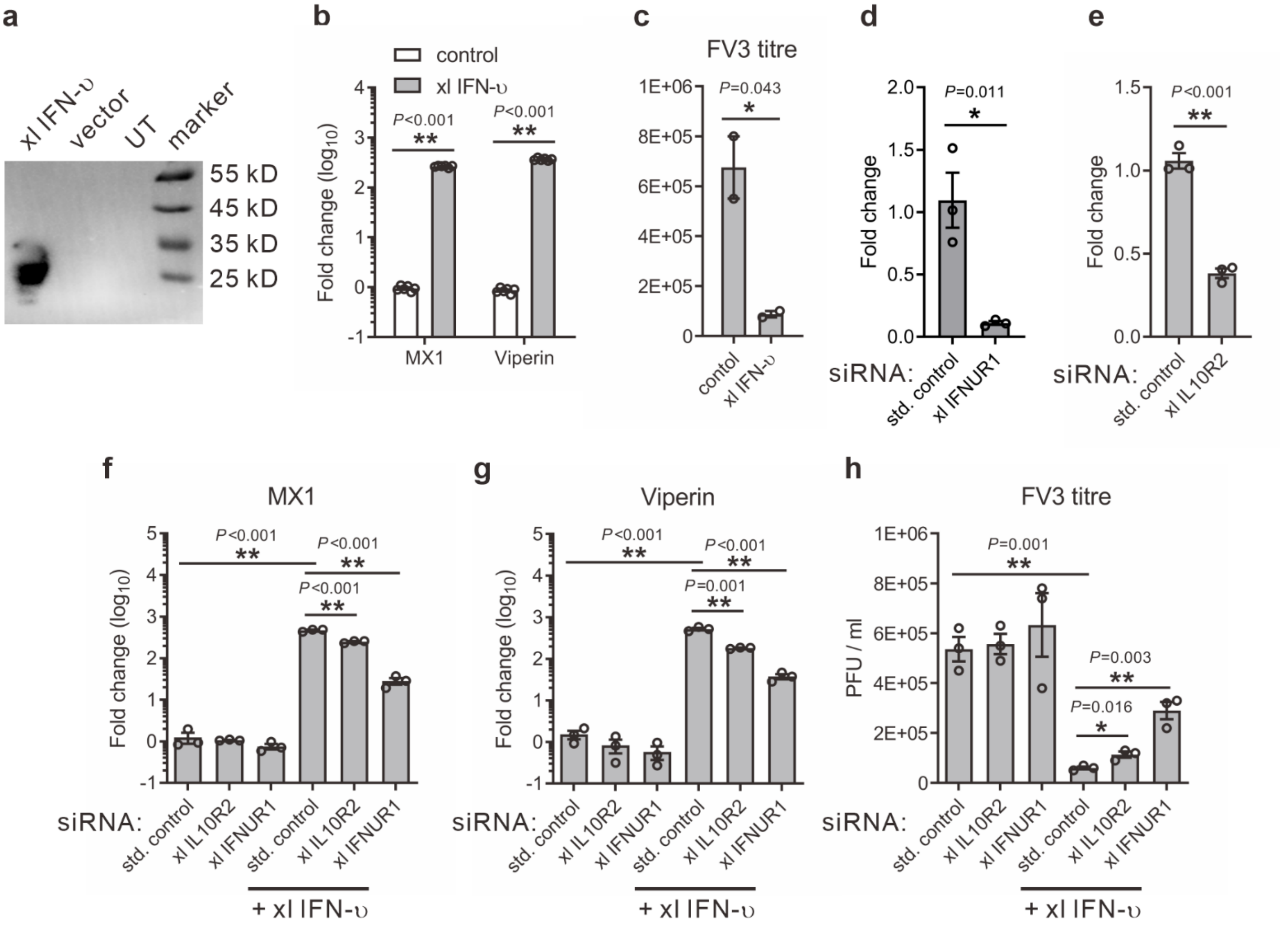

Supplementary Fig. 29. Antiviral function of *X. laevis* (xl, clawed frog) IFN-υ. (a) Western blotting detection of recombinant flag-tag-IFN-υ protein. Vector and UT represent supernatant media from empty plasmid-transfected and untreated HEK293T cells (negative control), respectively. Data represent two independent experiments. (b) Antiviral ISGs induced by xl-IFN-υ. A6 cells (1.0×10^{6}) were incubated with control medium and recombinant xl-IFN-υ containing supernatant produced in HEK293T cells for 10 hours. The mRNA expression level of ISGs (*mx1* and *viperin*) was determined by quantitative RT-PCR. The expression of the selected genes was normalized against *actb* and fold changes were calculated relative to control group. (c) Viral yields of FV3 reduced by xl-IFN-υ. Recombinant xl-IFN-υ and control medium were added to A6 cells (1.0×10^{6}) for 10 hours incubation. Subsequently, the cells were infected with FV3 at the multiplicity of infection (MOI) of 5, and viral titre was measured by plaque assay. The knockdown effects of *IFNUR1* (d) and *IL10RB* (e) siRNAs were detected by quantitative RT-PCR. (f-h) Effects on xl-IFN-υ-induced ISG expression and antiviral function by knockdown of *IFNUR1* and *IL10RB*. ISG expression and viral titre were determined by quantitative RT-PCR and plaque assay, respectively, after incubation with xl-IFN-υ and knockdown of *IFNUR1* or *IL10RB* in A6 cells. Data represent mean ± SEM from three independent experiments. The two-tailed Student's *t*-test was used to determine the statistical significance, * indicating $P < 0.05$, and ** $P < 0.01$.

Supplementary Table 1.

Comparison of class II cytokine receptors between zebrafish and human

Gene in human	Gene in zebrafish	Synonym in fish	Reference
IFNAR1	CRFB5	IFNAR1, IFNphiR2	2, 4
IFNAR2	CRFB1 (duplication)	IFNAR2-1, IFNphi1R1	2, 4
	CRFB2 (duplication)	IFNAR2-2, IFNphi2R1	2, 4
	CRFB3 (duplication, lost)	–	3
IFNGR1	CRFB13 (duplication)	IFNGR1-2	1, 4
	CRFB17 (duplication)	IFNGR1-1	1, 4
IFNGR2	CRFB6	IFNGR2	1, 4
IFNLR1 (IL28RA)	CRFB14?	–	
IL10R2 (IL10RB)	CRFB4	–	5, 6
IL10R1 (IL10RA)	CRFB7	IL10R1	5, 6
IL20R1 (IL20RA)	CRFB8	IL20R1	7, 8
IL20R2 (IL20RB)	CRFB16	IL20R2	8
IL22RA2 (IL22BP)	CRFB9	IL22BP	3
TF	CRFB10 (duplication)	TFa	1
	CRFB11 (duplication)	TFb	1
IL22RA1	CRFB14?	–	
?	CRFB12	IFNUR1	This study
?	CRFB15	–	

Supplementary Table 2.

IFNU and IFNUR1 genes in vertebrates

Species	Version	Gene	Coding Exons	Location or Accession Number
**Mammal**				
Ornithorhynchus anatinus (platypus)	Ornithorhynchus_anatinus-5.0.1	*IFNUR1*	7	NW_001794458.1: 8968090..8983283
		IFNU	5	NW_001794186.1: 7979635..7986399
Tachyglossus aculeatus (Australian echidna)	mTacAcu1.pri	*IFNU*	5	NC_052078.1:11991062...11998896
		IFNUR1	7	XP_038604300.1
**Bird**				
Anas platyrhynchos (mallard)	BGI_duck_1.0	*IFNUR1*	7	NW_004677101.1: 470841..475790
		IFNU	5	NW_004676454.1: 459194..462827
Gallus gallus (chicken)	GRCg6	*IFNUR1*	7	XP_015133867.1
		IFNU	5	CM000094.5: 11425652..11429077
Numida meleagris (helmeted guineafowl)	NumMel1.0	*IFNU*	5	NC_034410.1: 10798380...10801894
		IFNUR1	8	XP_021261921.1
Lagopus leucura (white-tailed ptarmigan)	USGS_WTPT01	*IFNU*	5	NW_024962334.1: 39653122...39649735
Cygnus olor (mute swan)	bCygOlo1.pri.v2	*IFNU*	5	NC_049170.1: 12780684...12784388
Falco naumanni (lesser kestrel)	bFalNau1.pat	*IFNU*	5	NC_054057.1: 49677902...49673758
		IFNUR1	7	XP_040470473.1
Tyto alba (Barn owl)	T.alba_DEE_v4.0	*IFNU*	5	NW_024881313.1: 28718759...28714675
		IFNUR1	7	XP_032851323.2
Athene cunicularia (burrowing owl)	athCun1	*IFNUR1*	7	XP_026704474.1
Coturnix japonica (Japanese quail)	Coturnix japonica 2.1	*IFNU*	5	NC_029517.1: 10239577...10242791
Corvus kubaryi (Mariana crow)	C.kubaryi_AGA036_p1.0	*IFNU*	5	NW_024581137.1: 47584542...47580504
Passer montanus (Eurasian tree sparrow)	ASM1480565v1	*IFNU*	5	NW_024356505.1: 32996006...32991891
Hirundo rustica (Barn swallow)	bHirRus1.pri.v2	*IFNU*	5	NC_053450.1: 144857819...144853799
Serinus canaria (Common canary)	cibio_Scana_2019	*IFNU*	5	NW_022041541.1: 11893959...11898360
Onychostruthus taczanowskii (white-rumped snowfinch)	ASM1759005v1	*IFNU*	5	NW_024500349.1: 14541092...14537005
Pipra filicauda (Wire-tailed manakin)	ASM394559v2	*IFNU*	5	NW_024104489.1: 135199...139326
Taeniopygia guttata (zebra finch)	bTaeGut1.4.pri	*IFNU*	5	NC_044213.2: 11693366...11697783
Lonchura striata domestica (Bengalese finch)	lonStrDom2	*IFNU*	5	NC_042567.1: 11945154...11949189
Catharus ustulatus (Swainson's thrush)	bCatUst1.pri.v2	*IFNU*	5	NC_046221.1: 11532927...11536898
Centrocercus urophasianus (Greater sage-grouse)	USGS_Curo_1.0	*IFNU*	5	NW_024884711.1: 36688616...36685237

Patagioenas fasciata monilis (band-tailed pigeon)	NIATT_ARIZONA	*IFNU*	5	LSYS01002888.1: 9223010...9226540
Limosa lapponica baueri (bar-tailed godwit)	Godwit_v1	*IFNU*	5	KZ505761.1: 18381...21963
Meleagris gallopavo (turkey)	Turkey_5.1	*IFNU*	5	NC_015016.2:16490175...16493601
Aquila chrysaetos chrysaetos (golden eagle)	bAquChr1.4	*IFNU*	5	NC_044006.1: 65964262...65960138
		IFNUR1	7	XP_040975145.1
Dromaius novaehollandiae (emu)	droNov1	*IFNU*	5	NW_020452870.1:438128...442620
Struthio camelus australis (African ostrich)	ASM69896v1	*IFNU*	5	NW_009271896.1: 4053371...4057941
Melopsittacus undulatus (budgerigar)	bMelUnd1.mat.Z	*IFNU*	5	NC_047527.1: 109446889...109450503
Columba livia (rock pigeon)	Cliv_2.1	*IFNU*	5	AKCR02000004.1: 11703526...11707123
		IFNUR1	7	XP_021145800.1
Apteryx mantelli mantelli (North Island brown kiwi)	AptMant0	*IFNUR1*	7	XP_013813805.1
**Reptile**				
Alligator mississippiensis (American alligator)	ASM28112v4	*IFNUR1*	7	XP_019337453.1
		IFNU	5	NW_017707901.1: 15171479..15177353
Anolis carolinensis (green anole)	AnoCar2.0	*IFNUR1*	7	XP_008119338.1
		IFNU	5	
Chrysemys picta bellii (painted turtle)	Chrysemys_picta_bellii-3.0.3	*IFNUR1*	7	XP_005301412.1
		IFNU	5	NC_024222.1: 3879779..3885334
Alligator sinensis (Chinese alligator)	ASM45574v1	*IFNU*	5	NW_005842092.1: 1021947...1027895
		IFNUR1	7	XP_025061739.1
Crocodylus porosus (Australian saltwater crocodile)	CroPor_comp1	*IFNU*	5	NW_017728886.1 20447234...20453945
		IFNUR1	7	XP_019391873.1
Gavialis gangeticus (Gharial)	GavGan_comp1	*IFNU*	5	NW_017729004.1:26645071...26650909
Mauremys reevesii (Reeves's turtle)	ASM1616193v1	*IFNU*	5	NC_052624.1: 24974939...24979882
		IFNUR1	7	XP_039345751.1
Trachemys scripta elegans (red-eared slider turtle)	CAS_Tse_1.0	*IFNU*	5	NC_048299.1:24091374...24096381
Chelonia mydas (Green sea turtle)	rCheMyd1.pri.v2	*IFNU*	5	CM026899.1:237610605...237605534
		IFNUR1	7	XP_027674429.2
Rafetus swinhoei (Swinhoes soft-shelled turtle)	ASM1942577v1	*IFNU*	5	CM033424.1:236101367...236095006
Gopherus evgoodei (Goodes thornscrub tortoise)	rGopEvg1_v1.p	*IFNU*	5	NC_044323.1:24464510...24469590
Chelydra serpentina (Common snapping turtle)	ASM1885937v1	*IFNU*	5	JAHGAV010000013.1:4478590...4473531
Pelodiscus sinensis Chinese_soft-shelled_turtle	PelSin_1.0	*IFNU*	5	NW_005851920.1: 952241...945889
Varanus komodoensis (Komodo dragon)	ASM479886v1	*IFNU*	5	SJPD01000020.1: 16360267...16369868
Podarcis muralis	PodMur_1.0	*IFNU*	5	NC_041323.1:16462237...16468403

(Common wall lizard)				
Zootoca vivipara (common lizard)	UG_Zviv_1	*IFNU*	5	NC_048614.1:14907892...14913259
Salvator merianae (Argentine black and white tegu)	HLtupMer6	*IFNU*	5	QVOM02000116.1: 43579960...43569936
Sceloporus undulatus (fence lizard)	SceUnd_v1.1	*IFNU*	5	NC_056527.1:15855374...15860670
Lacerta viridis (green lizard)	ASM90024590v1	*IFNU*	5	OFHU01004531.1: 280706...288104
Aspidoscelis marmoratus (Marbled whiptail)	AspMar1.0	*IFNU*	5	MTQE01002638.1: 36173146...36164211
Pogona vitticeps (central bearded dragon)	pvi1.1	*IFNU*	5	NW_018150717.1: 99684...92648
Sphenodon punctatus (tuatara)	ASM311381v1	*IFNU*	5	QEPC01013748.1: 8934031...8941185
Dermochelys coriacea (leatherback sea turtle)	rDerCor1.pri.v3	*IFNUR1*	7	XP_038271070.1
**Amphibian**				
Xenopus laevis (African clawed frog)	Xenopus_laevis_v2	*IFNUR1*	7	XP_018085133.1
		IFNU	5	MW924834
Rhinatrema bivittatum (two-lined caecilian)	aRhiBiv1.2	*IFNU*	5	LR584388.1: 112458824...112468536
Hymenochirus boettgeri (Congo dwarf clawed frog)	UCB_Hboe_1.0	*IFNU*	5	CM033474.1: 30452082...30458040
Xenopus tropicalis tropical_clawed_frog	UCB_Xtro_10.0	*IFNU*	5	MW924835
		IFNUR1	7	XP_002940450.1
**Fish**				
Latimeria chalumnae (coelacanth)	LatCha1	*IFNUR1*	7	XP_005994806.1
		IFNU	5	NW_005819652.1: 958231..964496
Lepisosteus oculatus (spotted gar)	LepOcu1	*IFNUR1*	7	XP_015193079.1
		IFNU	5	NC_023187.1: 39318848..39321474
Danio rerio (zebrafish)	GRCz11	*IFNUR1*	7	NP_001035443.1
		IFNU	5	MW547062
Callorhinchus milii (elephant shark)	Callorhinchus_milii-6.1.3	*IFNUR1*	7	XP_007890021.1
		IFNU1	5	NW_006890139.1: 2812074..2813536
		IFNU2	5	NW_006890139.1: 2822900..2825423
Astyanax mexicanus (cavefish)	Astyanax_mexicanus-2.0	*IFNUR1*	7	XP_007245285.2
		IFNU	5	NC_035906.1: 2208175..2210642
Polyodon spathula (Mississippi paddlefish)	ASM1765450v1	*IFNU*	5	NC_054536.1: 4955235...4957023
		IFNUR1	7	XP_041074472.1
Acipenser ruthenus (sterlet)	ASM1064508v1	*IFNU*	5	NC_048325.1:5321571...5323376
		IFNUR1	7	XP_033870228.1
Amia calva (bowfin)	AmiCal1	*IFNU*	5	CM030124.1: 40686713...40688751
Anguilla anguilla (European eel)	fAngAng1.pri	*IFNU*	5	NC_049204.1:33252085...33253765
		IFNUR1	7	XP_035288771.1
Anguilla rostrata (American eel)	ASM1855537v1	*IFNU*	5	CM031784.1:33382267...33383949

Anguilla japonica (Japanese eel)	ASM635231v1	*IFNU*	5	VDMF01024909.1:3871403...3873144
Megalops atlanticus (tarpon)	MATL_1.0	*IFNU*	5	CM032876.1: 12253353...12251769
Megalops cyprinoides (Indo-Pacific tarpon)	fMegCyp1.pri	*IFNUR1*	8	XP_036380572.1
Scleropages formosus golden arowana Asian bonytongue	ASM162426v1	*IFNU*	5	KV410874.1: 7361741...7363087
	fSclFor1.1	*IFNUR1*	7	XP_018610618.1
Heterotis niloticus (African bonytongue)	ASM1813684v1	*IFNU*	5	CM030905.1: 11171826...11170081
Alosa sapidissima (American shad)	fAloSap1.pri	*IFNU*	5	NC_055973.1:17031450...17032575
		IFNUR1	6	XP_041947255.1
Esox lucius (northern pike)	fEsoLuc1.pri	*IFNU*	5	NC_047589.1: 15815063...15813807
		IFNUR1	7	XP_028977103.2
Clupea harengus (Atlantic herring)	Ch_v2.0.2	*IFNU*	4	NC_045168.1: 18224806...18225835
Salmo salar (Atlantic salmon)	ICSASG_v2	*IFNU*	5	NC_027318.1: 16625066...16626185
Oncorhynchus mykiss rainbow trout	USDA_OmykA_1.1	*IFNU*	5	NC_048575.1: 25190713...25191829
		IFNUR1	7	NP_001117887.1
Chanos chanos (milkfish)	fChaCha1.1	*IFNU*	5	NC_044497.1: 16134686...16135897
Pygocentrus nattereri (red-bellied piranha)	fPygNat1.pri	*IFNU*	5	NC_051213.1: 10530231...10531859
Ictalurus punctatus (channel catfish)	IpCoco_1.2	*IFNU*	5	CM004414.1: 31816095...31812928
		IFNUR1	7	XP_017315324.1
Tachysurus fulvidraco (yellow catfish)	ASM372403v1	*IFNU*	5	NW_020847794.1: 279125...282142
		IFNUR1	7	XP_027034414.1
Cyprinus carpio (common carp)	ASM1834038v1	*IFNU*	5	NC_056595.1: 4563376...4564202
		IFNUR1	7	XP_018939179.1
Labeo rohita (rohu)	HRRL_Labeo_rohita_001	*IFNU*	5	JAFDUU010013128.1: 2689220...2688348
Colossoma macropomum (tambaqui)	Colossoma_macropomum	*IFNU*	5	NW_023494785.1: 8925353...8927409

Supplementary Table 3.
Primer sequences

Gene	Primer name	Sequence (5'-3')	Application
gapdh	qGAPDH-F	GTAACTCCGCAGAAAAGCCAGAC	qPCR
	qGAPDH-R	CAAAAGAAACTAACACACACACA	
gig2	gig2-F	AAGATGACTTGGCTGCTGGT	qPCR
	gig2-R	GATCTCGGCTGAGGTAGACG	
irf1	qDr-IRF1 F	GTGTCCAGAATGCGCATGCG	qPCR
	qDr-IRF1 R	GCCCACTGCTTGAACAGACA	
rsad2	zvig1.5	CGCCATCAGAGCATCCAGT	qPCR
	zvig1.3	TTCCACACCAACATCCAGAA	
mxa	qMX-F	AGACCATCCTCATTTCAGCAAACTCT	qPCR
	qMX-R	CAATCTTTTTGTTGAATGAATCCCCTG	
ifnu	IFNUsgRNA-F	TGTAATACGACTCACTATAAAGACGTTCATT GAGGCCCGTTTTAGAGCTAGAAATAGC	sgRNA amplification
	sgRNA-R	AAAAGCACCGACTCGGTGCC	
	IFNUmut-F	AGCAAAGAAAAAGCAGTGTGG	Detection of ifnu mutant
	IFNUmut-R	CCAGATTTGGATTAAACCAAC	
	DRIFNMR4	CGCTGAAGAATCTCTCGCAC	RACE PCRs
	DRIFNMR5	GCCGTCTGACAGAGCGTTTC	
	DRIFNMF3	CAACTGTGCGTTTGACTATGA	
	DRIFNMF4	CCACGACACCTCCTCAATACG	
	DRIFNMF3-RT	CAACTGTGCGTTTGACTATGAT	qPCR
	DRIFNMR3-RT	CTCATTTTCAACACCGACCGAG	
	DRIFNM-EXF4	CGGAATTCCCACCATGGCCTGGATTAGAATCGT	Plasmid construction
	DRIFNM-EXR6	GGGGTACCTTAACGTCGCCGTCTGACAG	
ifnur1	DR12-EXF4	CCGCTCGAGCGACCATGGTGATGACTGGTTTTCTGACCTGC	Plasmid construction
	DR12-EXR5	CCCAAGCTTTTATACTTCATATTTGTTATTATG	
	CRFB12.5	CTGAATGGCTTCCAGGACAA	Knockdown test
	CRFB12.31	TGATGACTGGCGGAAACACTGAT	
	crfb12-E3-sgRNA1-F	TAATACGACTCACTATAGCCTACAGTCAGGACCTCTGG GTTTTAGAGCTAGAAATAGC	sgRNA amplification
	crfb12-E3-F1	TCAGGGTAGCATTGTGACATCT	Detection of ifnur1 mutant
	crfb12-E3-R1	ATGGACCGTCTGACAAGTGAC	
crfb4	DRCRFB4-EXF1	CCGCTCGAGCGACCATGGTGATGTCCGCTTTTATCAGTTTTTC	Plasmid construction
	DRCRFB4-EXR1	CCCAAGCTTTTATATTATTTGCTGATTGTCC	
	DR4F3-MOtest	GAAGAATGTCCGCTTTTATCAG	Knockdown test
	DR4R3-MOtest	GCAACTTGTTTAGTCTCCACCC	
	Crfb4-B3	TAATACGACTCACTATAGGAGACTAAACAAGTTGCAG GTTTTAGAGCTAGAAATAGC	sgRNA amplification
	Crfb4-F2	GGAGGGGTTATGATCCAGTT	Detection of crfb4 mutant
	Crfb4-R2	TAAGAGTATGCTACTGAAAACGTAT	
crfb7	DR7-MOtest-F3	AACGAATCTTCCTGGTTGGTG	Knockdown test
	DR7-MOtest-R3	CTCACTGCAATAGATGTGATCG	
crfb8	DR8-MOtest-F4	GGCAGGACACGGATCTCCCAG	Knockdown test
	DR8-MOtest-R4	TGTTTGGGATGATCTTCCAGAG	
crfb14	DR14-MOtest-F5	TGAGTGCAGTGGTGACCGAAGT	Knockdown test
	DR14-MOtest-R5	CTTCGCCACAATACTCCACATC	
crfb15	CRFB15.5	AGTGGACGTCTCCACACAA	Knockdown test
	CRFB15.30	CTTCCAGACTGTTATTCTGGAT	

crfb16	DR16F5-MOtest	GATCGGAGAAGAAGAGGTTATG	Knockdown test
	DR16R2-MOtest	GTGTTGAAGCCCACCTGAATAG	
xl-ifnur1	XL-C12RNAi-F1	TATGTGCGTGTCCCTTCCTCTT	Knockdown test
	XL-C12RNAi-R1	TACAGTGCGATTCATTAGTCAG	
	XLCRFB12-F1	CTATGAAATGGCACAATGAGCAG	Gene cloning
	XLCRFB12-R1	GGATTGAGAAATATTGGGTCTG	
xl-il10r2	XLIL10RBLF2RT	AGCCTTCACTTGTCACCAGCAT	Knockdown test
	XLIL10RBLR2RT	ATGGCTTTGCTTAATTCTCCAG	
	XLIL10RB-F2L	AGCTCGGAGCACGTACCATTCC	Gene cloning
	XLIL10RB-R2L	GAGAAAAGCCCAGGCTGCCATTG	
	XLIL10RB-F1	GAGCACTTCTCATTCATTAAGCC	Gene cloning
	XLIL10RB-R1	GGGAAAAGCCGAGGCTGCAG	
xl-ifnu	XLIFNMLF1	AAATGCTGATGTCACAAATAC	Gene cloning
	XLIFNMLR1	TTTCAGGGTGAAGAAATGTTC	
	XLIFNMLEXF1	GGGGTACCCCACCATGCTGATGTCACAAATACTC	Plasmid construction
	XLIFNMLEXR2	GCTCTAGATCACTTGTCATCGTCATCCTTGTAGTCGGGTGAAG AAATGTTCATGTT	

Supplementary Table 4.

Morpholinos used in this study

Gene	GenBank ID	Sequence (5'-3')
crfb1	NM 001079681	CGCCAAGATCATACCTGTAAAGTAA
crfb2	NM_001077626	CTATGAATCCTCACCTAGGGTAAAC
crfb4	NM_001083868	GACATTGAAAAGTAACTTACTTCA
crfb5	NM_001034185	AGAGCGTATCCTCACCGTGTTTATC
crfb6	NM_001077627	AACTATCTCAAGTCCTTACCGATAT
crfb7	NM_001077625	ATGTCCACTGAAAAACCAACCTTAC
crfb8	NM_001079676	AAAATCCAAGGCAGTCACTCACTGT
crfb12	NM_001040353	ATACATATCTGAACTTACTGTCTCC
crfb13	GQ901864	TCTAAATGGTTTGTAGTTACCTGAG
crfb14	HM149255	CAGGAGAGATGATACTAACCTGTGA
crfb15	HM149256	AAAAACGGATTCACACTTACTGTGC
crfb16	HM149257	CTGAATCTGAAACGTGGGAACAATA
crfb17	GQ901865	TTAAACTAAATCGCCTTACCTTGTG
IFNphi1	NM_207640.1	CTGGTCCTCCACCTGTAATGCAATG
IFNGrel	NM_001020793.1	TTTCTGTGCTGTGAACCAAGTGATG
IFNG	NM_212864.1	TGAAGGCGTTCGCTAAAGTTAGAGT

Supplementary Table 5.

Genes used in sequence alignment and phylogenetic tree

Gene	Species, Gene Location or Accession Number
IFNAR1	human, NP_000620.2; chicken, NP_990190.1; zebrafish, NP_001029357.3;<br>grass_carp, AGZ04472.1; tropical_clawed_frog, XP_002942842.5;<br>platypus, XP_007667937.2; painted_turtle, XP_005283984.1; mouse, NP_034638.2
IFNAR2	chicken, NP_990189.1; mouse, NP_034639.2; human, NP_997468.1;<br>zebrafish_IFNAR2-1, NP_001073149.1; grass_carp_IFNAR2-1, AGW21650.1;<br>zebrafish_IFNAR2-2, NP_001071094.2; grass_carp_IFNAR2-2, AMT92200.1;<br>platypus, XP_028938459.1; tropical_clawed_frog, NP_001072391.1;<br>painted_turtle, XP_042711707.1;
IFNGR1	chicken, NP_001123859.1; human, NP_000407.1; anole, AGU16999.1;<br>zebrafish_IFNGR1-2, NP_001165063.1; goldfish_IFNGR1-2, ACV41809.1;<br>zebrafish_IFNGR1-1, NP_001129451.2; goldfish_IFNGR1-1, ACV41808.1;<br>platypus, XP_028913350.1; African_clawed_frog, XP_018120789.1; mouse, NP_034641.1
IFNGR2	human, NP_005525.2; chicken, NP_001008676.1; anole, AGL76447.1;<br>tropical clawed frog, CX465469.2 and EL666073.1; mouse, NP_032364.1;<br>zebrafish, NP_001071095.1; grass_carp, AMT92201.1; platypus, XP_028937875.1
IFNLR1 (IL28RA)	mouse, NP_777276.3; chicken, K9JA28.1; tropical clawed frog, NP_001165238.1;<br>human, NP_734464.1; anole, AHY86485.1; whale_shark, XP_020365568.1;<br>elephant_shark, XP_007892929.1; platypus, XP_028936126.1;
IL10R2 (IL10RB)	mouse, NP_032375.2; tropical clawed frog, NP_001165294.1; human, NP_000619.3;<br>chicken, NP_990188.1; anole, NP_001315154.1; zebrafish, NP_001077337.1 (crfb4);<br>grass_carp, AHJ11133.1; common_carp, XP_018965682.1; catfish, XP_017326115.1;<br>platypus, XP_028937522.1
IL10R1 (IL10RA)	mouse, NP_032374.1; human, NP_001549.2; chicken, NP_001034686.1;<br>tropical clawed frog, XP_002932948.2; alligator, XP_014382148.1; coelacanth, XP_014345361.1;
IL20R1 (IL20RA)	human, NP_055247.3; mouse, NP_766374.1; chicken, XP_419723.1;<br>zebrafish, NP_001073144.2; trout, NP_001118088.1
IL20R2 (IL20RB)	human, NP_653318.2; chicken, XP_015150082.1; tropical clawed frog, XP_004917828.1;<br>zebrafish, NP_001124447.1; catfish, XP_017351527.1; salmon, XP_013994619.1
IL22RA1	mouse, NP_839988.1; human, NP_067081.2; platypus, XP_001508953.2<br>pigeon, EMC78530.1; zebrafish_CRFB14, NP_001184131.1;<br>Sinocyclocheilus_IL22RA1L, XP_016374979.1; northern_pike_IL22RA1L, XP_010883684.1
IL10	human, NP_000563.1; chicken, NP_001004414.2; zebrafish, NP_001018621.2
IL22	human, NP_065386.1; chicken, NP_001186543.1
IL20	human, NP_061194.2; rat, NP_001137353.1
Type I IFN	human_IFNA2, NP_000596.2; human_IFNB, NP_002167.1; anole_IFN2, XP_016846269.1;<br>anole_IFN1, XP_008101700.1; chicken_IFNA, BAA83089.1; chicken_IFNB, NP_001020007.1;<br>tropical clawed frog_IFN1, CAO03085.1; tropical clawed frog_IFN2, CAO03086.1; carp_IFN3, ADI81045.1;<br>carp_IFN1, ADI81047.1; zebrafish_IFNphi1, NP_997523.1; zebrafish_IFNphi2, NP_001104552.1;<br>zebrafish_IFNphi3, NP_001104553.1; zebrafish_IFNphi4, NP_001155212.1;<br>chicken_IFNA3, XP_015132926.2; platypus_IFN8, XP_028910681.1; platypus_IFN7, XP_028910771.1;<br>platypus_IFN5, NP_001229647.1; platypus_IFN4, NP_001229640.1; platypus_IFN3, NP_001229639.1;<br>platypus_IFN1, NP_001229638.1; platypus_IFN6, NP_001229650.1; platypus_IFN2, NP_001229636.1;<br>green_anole_IFN5 XP_016846269.1; green_anole_IFN3 XP_008101701.1;<br>green_anole_IFN2 XP_008101702.1; green_anole_IFN1 XP_016846565.1;<br>green_anole_IFN4 XP_008101700.1;<br>African_clawed_frog_IFNX1 to IFNX22, ANQ43313.1-ANQ43334.1<br>African_clawed_frog_IFN1 to IFN7, ANQ43306.1-ANQ43312.1
Type II IFN	human_IFNG, NP_000610.2; chicken_IFNG, NP_990480.1; zebrafish_IFNG, NP_998029.1;<br>tropical clawed frog_IFNG, ABU54059.1; anole_IFNG, AGU16998.1; zebrafish_IFNGrel, NP_001018629.1

Type III IFN	platypus_IFNG, NC_041741.1: 16839696...16834260;<br>human_IFNL1, NP_742152.1; human_IFNL2, NP_742150.1; chicken_IFNL, NP_001121968.1;<br>tropical clawed frog_IFNL1, ACV32134.1; anole, AHY86484.1; platypus_IFNL3L, NC_041732.1:<br>platypus_IFNL4L, NC_041732.1: 3194807...3191703;<br>African_clawed_frog_IFNL1 to IFNL9, ANQ43335.1- ANQ43343.1;<br>African_clawed_frog_IFNLX1/ IFNLX2, ANQ43344.1/ANQ43345.1

Supplementary Table 6.

Sequences of oligonucleotides for RNAi and probe

Gene	Application	Sequence (5'-3')
clawed frog IFNUR1	knockdown	CCTCATTCTTACTGTTACT
clawed frog IL10RB	knockdown	CATCTGAAAGAGTACCTAA
zebrafish IFNUR1	probe	TCGAAACATTGAGCAAGGCTGATGGAACGCG[tttCATCATCATACATCATCAT]$_{30}$
	DIG labeled probe	DIG-ttATGATGATGTATGATGATGT

Supplementary References

1. Aggad D, et al. In vivo analysis of ifn-gamma 1 and ifn-gamma 2 signaling in zebrafish. *J. Immunol.* 185, 6774-6782 (2010).
2. Aggad D, et al. The two groups of zebrafish virus-induced interferons signal via distinct receptors with specific and shared chains. *J. Immunol.* 183, 3924-3931 (2009).
3. Levraud JP, et al. Identification of the zebrafish IFN receptor: Implications for the origin of the vertebrate IFN system. *J. Immunol.* 178, 4385-4394 (2007).
4. Zou J, Secombes CJ. Teleost fish interferons and their role in immunity. *Dev. Comp. Immunol.* 35, 1376-1387 (2011).
5. Wei H, Wang X, Zhang A, Du L, Zhou H. Identification of grass carp IL-10 receptor subunits: functional evidence for IL-10 signaling in teleost immunity. *Dev. Comp. Immunol.* 45, 259-268 (2014).
6. Huo HJ, Chen SN, Li L, Nie P. Functional characterization of IL-10 and its receptor subunits in a perciform fish, the mandarin fish, *Siniperca chuatsi*. *Dev. Comp. Immunol.* 97, 64-75 (2019).
7. Lutfalla G, Crollius HR, Stange-thomann N, Jaillon O, Mogensen K, Monneron D. Comparative genomic analysis reveals independent expansion of a lineage-specific gene family in vertebrates: The class II cytokine receptors and their ligands in mammals and fish. *BMC Genomics* 4, 29 (2003).
8. Stein C, Caccamo M, Laird G, Leptin M. Conservation and divergence of gene families encoding components of innate immune response systems in zebrafish. *Genome Biol.* 8, R251 (2007).
