# Supplementary material for: Identification and establishment of type IV interferon and the characterization of interferon-υ including its class II cytokine receptors IFN-υR1 and IL-10R2
Source: Nat Commun. 2022 Feb 22;13:999. doi: 10.1038/s41467-022-28645-6 (PMC8863823; doi:10.1038/s41467-022-28645-6)
Supplement: Supplementary file 3 — Description of Additional Supplementary Files [file 41467_2022_28645_MOESM3_ESM.pdf]

Description of Additional Supplementary Files

Supplementary Movie 1. Mating of the crfb4-/- zebrafish. The gene knockout fish (age of 4 months) were put into mating tanks with dividers to keep the male and female separated at night before observation, and the fish were mating in the next day morning.
