# Supplementary material for: Identification and establishment of type IV interferon and the characterization of interferon-υ including its class II cytokine receptors IFN-υR1 and IL-10R2
Source: Nat Commun. 2022 Feb 22;13:999. doi: 10.1038/s41467-022-28645-6 (PMC8863823; doi:10.1038/s41467-022-28645-6)
Supplement: Supplementary file 5 — Reporting summary [file 41467_2022_28645_MOESM5_ESM.pdf]

nature portfolio

Corresponding author(s): Pin Nie, School of Marine Science and Engineering, Qingdao Agricultural University, Qingdao, Shandong Province, 266109, China

Last updated by author(s): January 22, 2022

Reporting Summary

Nature Portfolio wishes to improve the reproducibility of the work that we publish. This form provides structure for consistency and transparency in reporting. For further information on Nature Portfolio policies, see our Editorial Policies and the Editorial Policy Checklist.

Statistics

For all statistical analyses, confirm that the following items are present in the figure legend, table legend, main text, or Methods section.

n/a	Confirmed	
☐	☒	The exact sample size (*n*) for each experimental group/condition, given as a discrete number and unit of measurement
☐	☒	A statement on whether measurements were taken from distinct samples or whether the same sample was measured repeatedly
☐	☒	The statistical test(s) used AND whether they are one- or two-sided<br>*Only common tests should be described solely by name; describe more complex techniques in the Methods section.*
☒	☐	A description of all covariates tested
☐	☒	A description of any assumptions or corrections, such as tests of normality and adjustment for multiple comparisons
☐	☒	A full description of the statistical parameters including central tendency (e.g. means) or other basic estimates (e.g. regression coefficient) AND variation (e.g. standard deviation) or associated estimates of uncertainty (e.g. confidence intervals)
☐	☒	For null hypothesis testing, the test statistic (e.g. *F*, *t*, *r*) with confidence intervals, effect sizes, degrees of freedom and *P* value noted<br>*Give P values as exact values whenever suitable.*
☒	☐	For Bayesian analysis, information on the choice of priors and Markov chain Monte Carlo settings
☒	☐	For hierarchical and complex designs, identification of the appropriate level for tests and full reporting of outcomes
☒	☐	Estimates of effect sizes (e.g. Cohen's *d*, Pearson's *r*), indicating how they were calculated

Our web collection on statistics for biologists contains articles on many of the points above.

Software and code

Policy information about availability of computer code

Data collection	The unannotated sequences (or the so-called intergenic region in the annotation version of Zv9) between annotated genes in zebrafish genome (assembly version: Zv9) were collected to carry out gene prediction or annotation using GENSCAN (http://hollywood.mit.edu/GENSCAN.html) and FGENESH (http://linux1.softberry.com/all.htm) programs with standard parameters. Other IFN and receptor sequences were downloaded from NCBI database or were collected by using BLAST (version: 2.2.19-2.2.31 and 2.3.0-2.12.0, https://blast.ncbi.nlm.nih.gov/Blast.cgi) with homologous sequence.
Data analysis	Multiple sequence alignments and PSIPRED program (http://bioinf.cs.ucl.ac.uk/psipred/) were used to predict alpha-helix regions. BLAST (version: 2.2.19-2.2.31 and 2.3.0-2.12.0, https://blast.ncbi.nlm.nih.gov/Blast.cgi) and FGENESH programs were used to analyze available genome sequences. For sequence analysis, Signal peptides and transmembrane regions were predicted using the program SignalP 3.0 Server (http://www.cbs.dtu.dk/services/SignalP-3.0/) and TMHMM Server v. 2.0 program (http://www.cbs.dtu.dk/services/TMHMM-2.0/), respectively. Protein structures were predicted by AlphaFold 2 and visualized by using Chimera (Version 1.15). Multiple sequence alignments were performed using the Clustal X program. Neighbor-joining (NJ) phylogenetic trees were constructed using MEGA 4.1/MEGA 7.0 package with 1,000 time repeat of bootstrap analysis. Data were analyzed statistically with the Student t test in SPSS 16.0 software. Primer 5.0 software was used to design PCR primers.

For manuscripts utilizing custom algorithms or software that are central to the research but not yet described in published literature, software must be made available to editors and reviewers. We strongly encourage code deposition in a community repository (e.g. GitHub). See the Nature Portfolio guidelines for submitting code & software for further information.

Data

Policy information about availability of data

All manuscripts must include a data availability statement. This statement should provide the following information, where applicable:

- Accession codes, unique identifiers, or web links for publicly available datasets
- A description of any restrictions on data availability
- For clinical datasets or third party data, please ensure that the statement adheres to our policy

The raw data for figures are provided in the Source data file. The complete CDS and amino acid sequence of ifnu in zebrafish, and also in African clawed frog have the GenBank accession numbers: MW547062.1 (https://www.ncbi.nlm.nih.gov/nuccore/MW547062.1/), UBY00466.1 (https://www.ncbi.nlm.nih.gov/protein/ UBY00466.1/), and MW924834.1 (https://www.ncbi.nlm.nih.gov/nuccore/ MW924834.1/), UBY00467.1 (https://www.ncbi.nlm.nih.gov/protein/ UBY00467.1/).

Field-specific reporting

Please select the one below that is the best fit for your research. If you are not sure, read the appropriate sections before making your selection.

☒ Life sciences ☐ Behavioural & social sciences ☐ Ecological, evolutionary & environmental sciences

For a reference copy of the document with all sections, see nature.com/documents/nr-reporting-summary-flat.pdf

Life sciences study design

All studies must disclose on these points even when the disclosure is negative.

Sample size	The sample size was determined according to the previous studies (PMID: 30389775, PMID: 32188757, PMID: 31914680, PMID: 21354627, PMID: 29102889 and PMID: 17371995), regarding animal experiments, quantitative PCR, emybro test and viral titre assay.
Data exclusions	For phylogenetic analysis, the predicted intracellular region sequences of class II receptor protein at C-terminus were removed manually since the extracellular region of receptor is mainly involved functionally in ligand interaction.
Replication	All assays are conducted with replication in at least two or three independent experiments with similar results to confirm reproducibility.
Randomization	Simple randomization was performed to assign embryos or cell lines were cultured in the same conditions and randomly divided into different treatment/conditions for experiments.
Blinding	During data collection, the investigators were not blinded to group allocation, but the sample IDs consist of species name and digital number, not the grouping information. During data analysis, sample IDs were provided to the investigators, but not the grouping allocation information.

Reporting for specific materials, systems and methods

We require information from authors about some types of materials, experimental systems and methods used in many studies. Here, indicate whether each material, system or method listed is relevant to your study. If you are not sure if a list item applies to your research, read the appropriate section before selecting a response.

Materials & experimental systems

n/a	Involved in the study	
☐	☒	Antibodies
☐	☒	Eukaryotic cell lines
☒	☐	Palaeontology and archaeology
☐	☒	Animals and other organisms
☒	☐	Human research participants
☒	☐	Clinical data
☒	☐	Dual use research of concern

Methods

n/a	Involved in the study	
☒	☐	ChIP-seq
☒	☐	Flow cytometry
☒	☐	MRI-based neuroimaging

Antibodies

Antibodies used	anti-GAPDH (1:1000, Cat#: 2058, mouse monoclonal antibody, 2D7, Dia-An Biotech, Inc), anti-IFNUR1 (1:200, Cat#: C0814, rat polyclonal antibody, Dia-An Biotech, Inc), anti-CRFB4 (1:200, Cat#: C0813, rat polyclonal antibody, Dia-An Biotech, Inc), anti-FLAG-tag (1:2000, Cat#: 2064, mouse monoclonal antibody, 2E5, Dia-An Biotech, Inc) and anti-digoxigenin-labelled peroxidase (anti-DIG-HRP, 1:500, Code: 200-032-156, mouse monoclonal antibody, HY-A1, Jackson ImmunoResearch)
Validation	Validation of all the antibodies was performed by the manufacturer, Dia-An Biotech, Inc (https://www.dia-an.cn/en/, service@dia-

an.com) and Jackson ImmunoResearch (https://www.jacksonimmuno.com/catalog/products/200-032-156)

Eukaryotic cell lines

Policy information about cell lines

Cell line source(s)	Ctenopharyngodon idella kidney (CIK, GDC0086), Epithelioma papulosum cyprini (EPC, GDC0174) and human embryonic kidney 293T (HEK293T, GDC0187) cells were purchased from the China Center for Type Culture Collection (CCTCC). The Xenopus laevis A6 cells (CCL-102) were obtained from American Type Culture Collection (ATCC).
Authentication	Cell lines were authenticated in this study based on monitoring in growth rate, cell morphology and gene expression.
Mycoplasma contamination	Mycoplasma contamination was not found in all detected cells.
Commonly misidentified lines (See ICLAC register)	No commonly misidentified lines was used in this study.

Animals and other organisms

Policy information about studies involving animals; ARRIVE guidelines recommended for reporting animal research

Laboratory animals	The zebrafish (AB strain) embryos and adults were purchased from China Zebrafish Resource Center (CZRC) and maintained at 28.5 C with 10 h dark/14 h light cycle. The male and female zebrafish (4-5 months old wild-type or mutant) were mated to generate offspring, and 1-5 day post-fertilization (dpf) larvae were sacrificed to perform quantitative PCR and infection experiments.
Wild animals	No wild animals were used in this study.
Field-collected samples	This study did not involve field-collected samples.
Ethics oversight	All animal experiments were conducted in accordance with the Guiding Principles for the Care and Use of Laboratory Animals and were approved by the Attitude of the Animal Ethical and Welfare Committee in Institute of Hydrobiology, Chinese Academy of Sciences.

Note that full information on the approval of the study protocol must also be provided in the manuscript.
